# Acute and persistent effects of oral glutamine supplementation on growth, cellular proliferation, and tight junction protein transcript abundance in jejunal tissue of low and normal birthweight pre-weaning piglets

**DOI:** 10.1371/journal.pone.0296427

**Published:** 2024-01-02

**Authors:** Johannes Schregel, Johannes Schulze Holthausen, Miriama Sciascia, Solvig Görs, Zeyang Li, Armin Tuchscherer, Elke Albrecht, Jürgen Zentek, Cornelia C. Metges

**Affiliations:** 1 Research Institute for Farm Animal Biology (FBN), Institute of Nutritional Physiology, Dummerstorf, Germany; 2 Department of Veterinary Medicine, Institute of Animal Nutrition, Freie Universität Berlin, Berlin, Germany; 3 Research Institute for Farm Animal Biology (FBN), Institute of Genetics and Biometry, Dummerstorf, Germany; 4 Institute of Muscle Biology and Growth, Research Institute for Farm Animal Biology (FBN), Dummerstorf, Germany; University of Life Sciences in Lublin, POLAND

## Abstract

Breeding for higher fertility has resulted in a higher number of low birthweight (**LBW**) piglets. It has been shown that LBW piglets grow slower than normal birthweight **(NBW)** littermates. Differences in growth performance have been associated with impaired small intestinal development. In suckling and weaning piglets, glutamine **(Gln)** supplementation has been associated with improved growth and intestinal development. This study was designed to examine the effects of oral Gln supplementation on growth and small intestinal parameters in LBW and NBW suckling piglets. At birth (day 0), a total of 72 LBW (1.10 ± 0.06 kg) and 72 NBW (1.51 ± 0.06) male piglets were selected. At day 1, litters were standardized to 12 piglets, and experimental piglets supplemented daily with either Gln (1 g/kg BW) or isonitrogenous amounts of Alanine **(Ala)** as control (1.22 g/kg BW) until day 12. Creep feed was offered from day 14 onward. Subgroups of piglets were euthanized at days 5, 12, and 26 for the analyses of jejunal morphometry, cellular proliferation, glutathione concentration and transcript abundance of tight junction proteins. From age day 11 to 21, Gln supplemented LBW (**LBW-Gln**) piglets were heavier than Ala supplemented LBW (**LBW-Ala**) littermates (*P* = 0.034), while NBW piglets were heavier until age day 26 compared to LBW littermates. Villus height was higher in LBW-Gln compared to LBW-Ala on age day 12 (*P* = 0.031). Sporadic differences among supplementation and birthweight groups were detected for jejunal cellular proliferation, cellular population and glutathione concentration, whereas age was the most dominant factor. These results show that Gln supplementation improved the growth of LBW piglets compared to LBW-Ala beyond the termination of Gln supplementation, but this was not associated with consistent effects on selected parameters of jejunal development.

## Introduction

In recent years, sow prolificacy has increased significantly and therefore litter inhomogeneity and the number of low birthweight **(LBW)** piglets [1]. Low birthweight piglets, in particular males, have impaired organ development, increased prevalence of disease and higher mortality [2]. These factors have led to animal welfare issues, and economic losses [3], as well as ethical discussions regarding modern pig production. At birth, LBW piglets have an immature small intestine **(SI)** [4] which has also been observed in underweight infants [5]. The development of the SI is of particular interest as it has not only digestive and immunological functions, but also creates a barrier against the environment [6]. This barrier is formed by mucus, and the epithelial cell layer of the SI, which are linked by tight junction proteins **(TJP)** [7]. To ensure the integrity of the SI, the epithelial cells of the villus undergo a highly dynamic proliferation, lineage-specific differentiation and apoptosis [8,9]. For this renewal of the epithelia cells, differentiated epithelial cells exit the crypt and migrate to the tip of the villi, where they eventually become apoptotic and are shed into the intestinal lumen [10,11]. Next to enterocytes, the entero-endocrine cells and the Paneth cells, goblet cells, form the majority of the mucus lining the gastrointestinal tract. [12–14]. There are three types of gel-forming mucins (neutral, acidic and mixed) secreted by goblet cells, characterized based on their affinity for various dyes used for histological staining [14]. It is believed that neutral mucins are secreted by immature goblet cells, whilst acidic mucins are secreted by mature goblet cells [14,15]. Several factors such as age, local microbial population and availability of amino acids influence the abundance and the activity of goblet cells but the complex interaction of these factors is not completely understood [15–18]. In the colon of fattening pigs pathogenic *Brachyspira hyodysenteriae* interact with goblet cells and create an optimal environment for other pathogenic bacteria [19]. However, in humans inflammatory diseases such as ulcerative colitis are associated with depletion of goblet cells [18,20].

It has been reported that neonatal LBW piglets have impaired intestinal barrier function, which is accompanied by a lower abundance of TJP, and may explain their higher disease susceptibility [8]. Several nutritional strategies have been developed to overcome impaired growth and gut development, including amino acids **(AA)** supplementation [1]. The AA glutamine **(Gln)** is of particular interest because it is rapidly metabolized by enterocytes of the SI, and one of the most abundant AAs in sow’s milk [21]. *In vitro* studies using IPEC-cells showed positive effects of Gln on cellular proliferation, and barrier function [7]. Furthermore, Gln can be a precursor of glutathione which is a key component for the antioxidative defense [22]. Glutamine supplementation has been investigated in piglets, but most studies used weaned piglets. Suckling piglets are nutritionally and metabolically distinct compared to weaner piglets. Under commercial conditions piglets are weaned at an age of 3–4 weeks, while the development of SI is not yet complete [23]. The intestine of neonatal piglets is adapted to digest milk-based diets very efficiently but not grain-based diets because they have not achieved their full digestive capacity compared to adult pigs due to the lack of activities of disaccharidases such as sucrase, isomaltase or maltase in the SI mucosa which increase later [23,24]. Due to early and abrupt weaning practices piglets show low feed intake, growth arrest due to villus atrophy and loss of absorptive capacity and activity, and higher susceptibility to enteral infection [25–28]. Weaning is also associated to stress as piglets are separated from their mothers, transported to other farms and mixed with piglets from other litters which has been linked with higher intestinal barrier permeability [26–28]. The observation that Gln is beneficial in weaned piglets may indicate that Gln supplementation is particularly effective under challenging conditions [29,30]. We and others have previously shown that Gln supplementation at 1 g/kg BW per day increased the growth rate of suckling piglets [21,22,31]. However, it is not clear whether a higher rate of growth is associated with persistent changes in intestinal development. From a nutritional perspective, the later suckling period is interesting because under current farming conditions the piglets are offered solid creep feed, which initiates adaptation to grain-based diets [26]. This adaptation process is associated with changes in intestinal morphology, cellular population, and enzyme activity [1].

We have previously shown that Gln supplementation from age 1 to 12 days has acute beneficial effects on the growth of LBW piglets while jejunal parameters showed little differences [31,32]. We hypothesize that Gln might have positive persistent effects on growth and jejunal development, potentially alleviating the subsequent negative effects of current weaning practices. Therefore, in the present study we investigated whether the growth advantage for LBW piglets observed until age day 12 persisted beyond the period of acute Gln supplementation. Furthermore, we studied whether Gln supplementation affects the development of jejunal morphology, cellular proliferation, oxidative capacity and TJP transcript abundance before and after introducing piglets to creep feed consumption starting on age day 14.

## Material and methods

In the current study the focus is on the comparison of piglets at age 12 and 26 days, to investigate whether the effects of Gln persist after cessation (day 12) of Gln supplementation. Therefore, previously published data on zootechnical measures, jejunal AA concentrations, jejunal cellular population and jejunal morphology of age day 5 and 12 piglets [31,32] were combined with the data from the day 26 group and re-analyzed. New data on tight junction protein mRNA abundances, and cell proliferation are presented for age groups 5, 12, and 26 days.

### Animals, experimental design, and sample collection

All experimental procedures were approved by the licensing authority State Office for Agriculture, Food Safety and Fishery, Mecklenburg-Western Pomerania, Germany (permission No. 7221.3-1-026/16), and performed according to the German Animal Welfare Act following the Directive 2010/63/EU (European Convention for the Protection of Vertebrate Animals used for Experimental and Other Scientific Purposes). The experiment was performed at the experimental pig facility of the Research Institute for Farm Animal Biology (FBN), Dummerstorf, Germany, and has been described in detail [31]. Briefly, piglets were selected from gilt litters and standardized to 12 piglets within 24 h after birth, containing at least one littermate pair with one LBW (0.8–1.2 kg) and one normal birthweight (**BiW**) (**NBW**; 1.4–1.8 kg) male piglet. The selected LBW piglet population had a BiW below the lowest BiW quartile of the FBN experimental pig herd [33] and a significantly lower probability of survival compared to piglets with a birthweight > 1.18 kg [34]. The day after birth (day 1), LBW and NBW littermates were allocated to Gln (1 g/kg BW per day), or alanine (**Ala**) (1.22 g/kg BW per day; isonitrogenous control to Gln) supplementation groups (**Suppl**). Alanine supplementation was used in the control groups to compensate for the additional nitrogen supplied to the piglets via glutamine. Piglets were supplemented three times daily (07:00, 12:00, 17:00 h) until age day 12 (morning). Thus, the four treatment groups (*n* = 12/group) LBW-Gln, LBW-Ala, NBW-Gln and NBW-Ala were each studied in three different age groups (**Age**) each, i.e. day 5 (*n* = 48), 12 (*n* = 48), and 26 (*n* = 48), with 5 and 12 day groups examining the effects during supplementation, and the day 26 group, to study potential persistent effects beyond the supplementation period. However, the majority of the measurements in the day-5 age group has been reported previously [31,32].

Creep feed (**S1 Table**) was provided to the entire litter (experimental and non-experimental piglets) from age day 14 onwards (day 26 group). Zootechnical measurements including rectal temperature, crown-rump length (**CRL**), and abdominal circumference (**AC**) were recorded at birth and on age day 12, 14, 21, and 26, and the body mass index (**BMI**) was calculated as described [31]. Piglets were euthanized by exsanguination on age day 5, 12 or 26. Before euthanasia piglets were stunned with a bolt gun (age day 5) or electrically stunned (age day 12 and 26) according to legally prescribed procedures. Two hours (h) prior to euthanasia piglets were supplied with glutamine (0.33 g/kg BW) or alanine (0.41 g/kg BW), respectively, and one h before, piglets were injected *i*.*p*. with bromodeoxyuridine (**BrdU**) (12 mg/kg BW; Roche Diagnostics GmbH, Mannheim, Germany). Jejunal tissue was sampled as reported [32].

### Measurement of milk intake

Milk intake over 24 h was measured from age day 25 to 26 using the deuterium oxide (**D**_**2**_**O**) isotope dilution method [31]. Piglets were injected *i*.*p*. with D_2_O (0.2 mL/kg BW, 70 atom % D diluted to 20% in sterile physiological saline solution; B. Braun SE, Melsungen, Germany) 24 h before euthanasia. Afterwards piglets were isolated for one h to prevent suckling and to guarantee that the body water pool and D_2_O had equilibrated. One h (day 25) and 24 h (day 26, at euthanasia) after D_2_O injection blood samples were taken and its D enrichment was determined to calculate milk intake [31].

### Jejunal morphometry and immunohistochemistry

#### Assessment of morphometry and quantification of goblet cells.

Histo-fixed jejunum samples were cut into 5 μm sections using a microtome (Type 1400 Fa. Leitz Wetzlar, Germany). Jejunal mucosa morphometry, number and mucin type of goblet cells were determined using paraformaldehyde fixed tissue, and Alcian blue pH 2.5 periodic acid Schiff staining, as previously described [32]. A microscope (Photomicroscope BX43F, Olympus, Tokyo, Japan) equipped with a digital camera (Olympus DP72, Tokyo, Japan) was used and images were analyzed with the cellSens imaging software (v. 1.4, Olympus). Five crypts and corresponding villi were randomly selected from at least four sections per animal. The distance from the crypt mouth to the tip of the villus was defined as villus height **(VH)**. Whereas the distance from base of the crypt to the crypt mouth was specified as crypt depth **(CD)**.

#### Quantification of lymphocytes, IgA positive cells, and bromodeoxyuridine incorporation.

The quantification of immunoglobulin A (**IgA**) and cluster of differentiation 3 (**CD3**)-positive intraepithelial lymphocytes was performed as reported [32] using paraformaldehyde fixed tissue and immunohistochemistry. For IgA quantification slides were incubated at 4° C overnight with the following primary antibody: goat anti-porcine IgA 1:4000 (NB724, Novus Biologicals, Abingdon, UK). Immune reaction was visualized using a 3,3´-diaminobenzidine chromogen solution (DAB Substrate kit, Vector Laboratories). IgA-positive lymphocytes were quantified in jejunal lamina propria according to Waly et al. (2001) [35].

For quantification of CD-3 positive lymphocytes slides were incubated with a primary antibody (mouse anti porcine CD3 epsilon, CAT NO 4510–01, Southern Biotech) overnight at 4°C. The primary antibody was visualized using an indirect two-step method (Mouse and Rabbit Specific HRP/DAB IHC Detection Kit, ab236466, ABCAM). A horseradish peroxidase (HRP)-conjugated micropolymer (goat anti-rabbit HRP conjugate, 58009, ABCAM) was used as secondary antibody. All immunohistochemistry was performed according to published procedures [36]. For evaluation of the stained sample, a double-blind quantification of CD3-positive intraepithelial lymphocytes was carried out. Only intact and complete villi (two sections per animal, five villi per section) were evaluated. The number of positive lymphocytes was expressed per 100 enterocytes.

Bromodeoxyuridine is an analogue of the nucleoside thymidine and BrdU incorporation is a measure of cellular proliferation. Bromodeoxyuridine incorporation was determined by Alexa Fluor 488 BrdU labelling [37], with the following modifications. Frozen jejunal tissue (-80°C) was cryosectioned into 5 to 6 slices per animal (5–8 μm, Leica CM3050 Cryostat Microtome; Leica, Nussloch, Germany), with the following BrdU-labelling pre-treatment steps: 1) Fixation (4% paraformaldehyde, 20 min, Roti-Histofix, Roth, Karlsruhe, Germany); 2) Permeabilization (0.1% Triton X-100 in phosphate-buffered saline (**PBST**), 10 min); 3) DNA denaturation (2 M HCl, 37°C, 60 min); blocking of unspecific bindings of the secondary antibody (10% goat serum, 15 min, 16120–099, Thermo Fisher Scientific, Schwerte, Germany).

The primary antibody was a monoclonal mouse anti-BrdU (1170376, Roche Diagnostics AG, Rotkreuz, Switzerland; 1∶100 dilution in PBST containing 2% goat serum) whilst the secondary antibody was an Alexa Fluor 488 goat anti-mouse IgG (A11059, Thermo Fisher Scientific; 1∶500 dilution in PBST). To visualize unlabeled nuclei, sections were counterstained 5 min with propidium iodide (5 μg/mL, no. P4170, Sigma-Aldrich). Sections were placed under the cover of ProLong Antifade mounting medium (Thermo Fisher Scientific) and coverslips. A Nikon Microphot SA fluorescence microscope (Nikon, Düsseldorf, Germany) equipped with a CC-12 color camera (OSIS, Münster, Germany) was used to detect immunofluorescence of BrdU and propidium iodide-stained nuclei. Images were analyzed with Cell^F image analysis software (OSIS). The total area of BrdU-labelled crypt and villi cell nuclei (labelled green) as well as the total area of all nuclei (labelled red) were determined in 4 randomly chosen fields per slice and 20 fields (10 crypt and 10 villi) per animal. The area of all detected objects was measured in red and green channels, respectively. The ratio between the BrdU-labelled area and total nuclei area was determined as a measure of cell proliferation.

### Localization of tight junction proteins in jejunal tissue

Immunohistochemistry was used to visualize the location of the following target proteins: Tight junction protein 1 (**TJP1**) and tight junction protein 2 (**TJP2**), claudin-4 (**CLDN4**) and occludin (**OCLN**). Jejunal tissue frozen in OCT matrix (OCT Embedding Matrix, Roth, Karlsruhe, Germany), was cryo-sectioned to obtain 5 to 6 slices per animal (10 μm, Leica CM3050 Cryostat Microtome; Leica, Nußloch, Germany). For TJP1 and TJP2 detection, slides were stored for 15 min in 4% paraformaldehyde, (Roti-Histofix, Roth) for fixation, whilst the slides for CLDN4 and OCLN detection were fixed for 10 min in methanol at -20°C. Slides were then rinsed three times in phosphate-buffered saline (PBS) for 5 min. Unspecific binding of the secondary antibodies was blocked by incubating slides with goat serum for 15 min (10% goat serum in PBS, 16120–099, Thermo Fisher Scientific).

The following primary antibodies were used for overnight incubation at 4°C in a humidity chamber: TJP1 (rat, SC-33725, Santa Cruz, California, USA, 1∶50 dilution in PBST containing 2% goat serum), TJP2 (rabbit, CS-2847, Cell Signaling Technology, Danvers, Massachusetts, USA, 1∶50 dilution in PBS containing 2% goat serum), CLDN4 (mouse, 3E2C1, Thermo Fisher Scientific, Waltham, Massachusetts, USA, 1:200 dilution in PBST containing 0.5% goat serum), and OCLN (mouse, SC-133256, Santa Cruz, 1:200 dilution in PBS containing 0.5% goat serum). Slides were then rinsed three times in PBS for 10 min, followed by incubation with the respective secondary antibody in a dark humidity chamber (for TJP1 Alexa Flour 488, goat anti rat IgG, 1:1000 dilution in PBST; for TJP2 Alexa Flour 488 goat anti rabbit IgG, 1:1000 dilution in PBS; for OCLN and CLDN4 Alexa Flour 594 goat anti mouse IgG, 1:1000 dilution in PBS). After the slides were washed three times for 10 min in PBS, they were incubated with 1 μg/ml Hoechst 33258 (Sigma-Aldrich, Munich, Germany) for 5 min. Afterwards, the slides were rinsed twice with PBS for 5 min and once with ultra-pure water. Then, slides were mounted with coverslips using ProLong Antifade mounting medium (Thermo Fisher Scientific). Jejunal tight junction proteins (TJP1, TJP2, OCLN, CLDN4) were visualized in cross sections using Nikon Microphot SA fluorescence microscope (Nikon, Düsseldorf, Germany) equipped with a CC-12 color camera and CellˆF image analysis software (OSIS, Münster, Germany).

### Quantification of jejunal transcript abundances connected to barrier function, apoptosis and cellular proliferation

#### Purification of RNA and cDNA synthesis.

Isolation and purification of RNA, and cDNA synthesis were performed as described [32]. Briefly, RNA was extracted from frozen jejunal tissue (80–120 mg) using the trizol method, according to the manufacturer’s instructions (peqGold TriFast; VWR International, Hanover, Germany). A microcolumn-based system (Qiagen, Hilden, Germany) was then used to purify the isolated RNA. The RNA integrity number ranged from 6.9 to 9.9 (mean 8.9 ± 0.7) and was measured using a Bioanalyzer 2100 and RNA 6000 Nano kit (Agilent Technologies, Waldbronn, Germany). The SensiFast cDNA Synthesis Kit (Bioline, Berlin, UK) was utilized to reverse transcribe purified RNA (500ng) to cDNA following the manufacturer’s instructions.

#### Primer design, real time PCR assay and data evaluation.

Primers were designed, manufactured, and verified [32]. Primer details are presented in the supporting information **(S2 Table)**. The real time PCR assays were operated on the LC 96 system (Roche Diagnostics, Mannheim, Germany). Setup and reaction conditions were as published [32]. The LinRegPCR v 2014.5 software was used to calculate PCR efficiency and quantification cycle values [38]. Quantification cycle values and average PCR-efficiency are reported in the supporting information (**S2 Table**). The GeNorm applet from qBASEplus was applied to select the reference genes from 6 candidates (**S2 Table**) with the most stable expression across BiW and Suppl. In the 5-day group, beta actin (ACTB), ribosomal protein S18 (RPS18), and DNA topoisomerase 2-beta (TOP2B) were chosen, while in the 12-day group peptidylprolyl isomerase A (PPIA) and ribosomal protein S18 (RPS18) were selected. In the 26-day group peptidylprolyl isomerase A (PPIA) and hypoxanthine phosphoribosyltransferase 1 (HPRT) were most stably expressed. Target gene mRNA abundances were normalized using the qBASEplus software (CellCarta, Antwerpen, Belgium) and the selected reference genes. The qBASEplus software was used to convert quantification cycle values into Log-CNRQ (Calibrated Normalized Relative Quantity) values, considering amplification efficiencies, normalization factors, and inter-run variations. The Minimum Information for Publication of Quantitative Real-Time PCR Experiments (MIQE) guidelines were followed to report all data [39].

### Concentration of amino acids and glutathione in jejunal tissue

#### Amino acid concentration in plasma, and jejunal tissue.

At euthanasia blood samples were collected in K-EDTA (Sarstedt, Germany) tubes and centrifuged at 1,576 g for 20 min (4°C) to generate plasma and stored at– 80°C. Plasma samples were prepared for HPLC analysis and free AAs concentration was measured as previously published [31], using HPLC and a 250 × 4 mm Hyperclone ODS (C18) 120Å column, (Phenomenex, Aschaffenburg, Germany).

Enzymatic hydrolysis was performed to determine the concentration of Gln and other protein-bound AA to calculate total AAs in jejunal tissue as described [32]. The concentrations of free and protein-bound AAs were measured using a HPLC equipped with 5 μm C18 columns, 250 x 4 mm HyperCloneTM 120 Å or 250 x 4.6 mm Gemini ® 110 Å (both Phenomenex). To calculate the concentration of protein bound AAs, the concentration of free AA were subtracted from the concentration of total AAs [32]. Additionally, a BCA reagent (Biorad Laboratories, Feldkirchen, Germany) was used to measure total protein concentration in jejunal tissue photometrically.

#### Glutathione in jejunal tissue.

The quantification of glutathione in jejunal tissue was performed as described [40]. Frozen jejunal tissue (30 mg) was mixed with 60 μL of iodoacetic acid (Sigma Aldrich, Munich, Germany) (25 mg/mL in 0.5 M bicine buffer pH 9.0). Subsequently, the samples were diluted with 600 μL ultrapure water. A HPLC equipped with 5 μm C18 columns, 250 x 4 mm HyperCloneTM 120 Å or 250 x 4.6 mm Gemini® 110 Å (both Phenomenex) was used to measure the concentration of glutathione in jejunal tissue.

### Statistical evaluation and data presentation

Previously published zootechnical data, plasma and jejunal tissue free and protein-bound AA concentrations, jejunal morphometry and cellular populations of the 5- and 12-day group piglets [31,32] were combined with data from the 26-day group and re-evaluated as a single joint dataset.

The necessary sample size was computed with CADEMO for Windows ANOV version 4.03 (2000; BioMathGmbH, Rostock, Germany), based on a two or three level factor combination of (1) supplementation (Ala, Gln), (2) birthweight (LBW, NBW), and (3) age group (5, 12, 26 days). Alpha and beta levels were defined as α = 0.05, and β = 0.20, respectively. The group size for each experimental procedure was *n* = 12, unless otherwise indicated. Statistical analysis of the data was performed in SAS (version 9.4; SAS Institute Inc., Cary, NC, USA) via Analysis of Variance (ANOVA), with normal distribution assessed using the Shapiro-Wilks test, and the model selection based on Akaike’s information criterion [41]. For all models pig was the experimental unit. Model 1, used for the evaluation of average daily body weight gain, jejunal morphology, histological parameters, AA concentration, cellular proliferation and real time PCR assessment, was a MIXED model that contained the fixed effects Age group (5, 12, 26 days), Suppl (Gln, Ala), BiW (LBW, NBW), the Age × Suppl × BiW interaction and gilt as a random effect to model the possible dependence of littermates from the same gilt. Model 2, used for the assessment of CRL, AC, BMI, and rectal temperature, was the same as Model 1 but also included a repeated measures statement and the fixed effect Age at measurement (days 0, 5, 7, 12, 14, 21, 26). The repeated measures statement accounted for measurements taken in the same animal at different ages using the SUBJECT = animal option to define the blocks of the block-diagonal residual covariance matrix and the TYPE = VC option to define their covariance structure. Model 3, assessing BW was the same as Model 2, but contained the fixed effect of Age at measurement (days 0–12, 14, 21, 26) and a linear spatial covariance structure (TYPE = SP (LIN)). If *P* < 0.05 (Tukey–Kramer test) differences were considered significant. Data are shown as least square means **(LSM)** and standard error of the mean **(SEM)**. Two animals with their respective supplementation partners were removed from the statistical analysis due to growth arrest.

## Results

### Zootechnical measurements

Supplementation affected AC, while BiW had an influence on BW, CRL, AC, BW gain, and BMI. The factor Age affected all parameters measured while the interaction of Age × Suppl × BiW affected BW only (Table 1). In LBW-Gln compared to LBW-Ala piglets, BW was higher from age days 12 to 21 (*P* = 0.034). Furthermore, AC was higher at age day 26 in NBW-Gln compared to NBW-Ala piglets (*P* = 0.042). Throughout the study, BW gain was higher in the NBW compared to LBW littermates independent of supplementation (*P* < 0.001). Consequently, BW was higher in NBW compared to LBW at each time point (*P* < 0.001). Piglets with NBW were born with a higher AC, BMI and CRL compared to LBW piglets (*P* = 0.001). During the entire trial (birth to 26 days of age), NBW-Ala had a higher AC and CRL compared to LBW-Ala (*P* = 0.048), whilst CRL was higher in NBW-Gln than in LBW-Gln on age day 14 and 26 (*P* = 0.043), and AC was higher in NBW-Gln compared to LBW-Gln on age day 12 and 26 (*P* < 0.001). Body mass index was higher in NBW-Ala compared to LBW-Ala at age day 26 (*P* = 0.040). The parameters rectal temperature (*P* = 0.179) and milk intake (*P* = 0.061) were not different among BiW and Suppl groups (Table 1).

**Table 1:** Acute (age day 12) and persistent (age days 13–26) effects of an early life (day 1–12) glutamine (Gln) or alanine (Ala) supplementation on zootechnical measurements in low (LBW) and normal birthweight (NBW) suckling piglets.

		Ala		Gln			*P*-Value ^1^			
Item	Age (day)	LBW	NBW	LBW	NBW	SEM	Suppl	BiW	Age	Interaction ^2^
BW (kg)	birth	1.10 ^d^	1.53 ^c^	1.09 ^d^	1.50 ^c^	0.061	0.184	<0.001	<0.001	<0.001
	12	3.06 ^b^^,^ ^d^	3.96 ^c^	3.25 ^a^^,^ ^d^	4.02 ^c^	0.067				
	14	3.33 ^b^^, d^	4.34 ^c^	3.54 ^a^^,^ ^d^	4.41 ^c^	0.075				
	21	4.75 ^b^^,^ ^d^	6.10 ^c^	5.15 ^a^^,^ ^d^	6.15 ^c^	0.092				
	26	5.88 ^d^	7.32 ^c^	5.94 ^d^	7.30 ^c^	0.097				
BWG (g/day)	6–12	165 ^c^	200 ^d^	179 ^c^	204 ^d^	8.36	0.303	<0.001	-	-
	13–26	186 ^c^	223 ^d^	187 ^c^	224 ^d^	10.9	0.963	<0.001	-	-
AC (cm)	birth	21.7 ^d^	24.5 ^c^	22.0 ^d^	24.2 ^c^	0.371	0.002	<0.001	<0.001	0.980
	12	32.4 ^d^	34.1 ^c^	32.4 ^d^	35.4 ^c^	0.617				
	14	32.3 ^d^	35.4 ^c^	33.8	35.2	0.641				
	21	37.2 ^d^	39.4 ^c^	39.1	40.8	0.706				
	26	41.0 ^d^	42.6 ^b^^,^^c^	41.2 ^d^	44.4 ^a^^,^ ^c^	0.641				
CRL (cm)	birth	22.6^d^	24.8 ^c^	22.4 ^d^	24.9 ^c^	0.416	0.630	<0.001	<0.001	0.521
	12	31.5^d^	34.0 ^c^	32.9	34.4	0.665				
	14	33.2^d^	36.1 ^c^	32.9 ^d^	36.3 ^c^	0.684				
	21	38.6 ^d^	41.7 ^c^	38.9	40.7	0.752				
	26	41.6 ^d^	44.2 ^c^	41.0 ^d^	44.4 ^c^	0.684				
BMI (kg/m^2^)	birth	20.9 ^d^	24.1 ^c^	21.8 ^d^	24.0 ^c^	0.707	0.410	<0.001	<0.001	0.999
	12	31.7	33.9	30.9	33.3	1.12				
	14	30.0	33.1	31.2	33.1	1.27				
	21	32.5	34.6	34.1	36.8	1.27				
	26	34.2 ^d^	37.6 ^c^	35.2	37.2	1.15				
Rectal temperature (C°)	birth	37.3	37.5	37.3	37.4	0.184	0.289	0.772	<0.001	0.730
	12	38.4	38.0	37.8	38.4	0.297				
	14	39.0	39.0	38.6	38.8	0.308				
	21	39.2	39.2	39.0	38.9	0.340				
	26	39.1	38.8	38.3	38.4	0.308				
Milk intake (g/kg BW day)	26	199	144	144	174	22.3	0.578	0.551	.	.

Values are LSM ± SEM; day 0–5 *N* = 36/group, day 6–12 *N* = 24/group, day 14–26 *N* = 11/group.

^1^ ANOVA F-Test; none if the interactions (Suppl x BiW, Suppl x Age, or BiW x Age) were significant (*P* > 0.05).

^2^ Interaction Suppl x BiW x Age.

^a,b^ Labeled LSM in a row within one Birthweight group and one Age group without a common letter differ, *P* < 0.05 (Tukey-Kramer test).

^c,d^ Labeled LSM in a row within one Supplementation group and one Age group without a common letter differ, *P* < 0.05 (Tukey-Kramer test).

Abbreviations: AC = Abdominal circumference, BiW = Birthweight, BMI = Body mass index, BW = Bodyweight, BWG = Bodyweight gain, CRL = Crown-rump length, Suppl = Supplementation.

### Jejunal morphometry and goblet cell abundance

**Fig 1:**
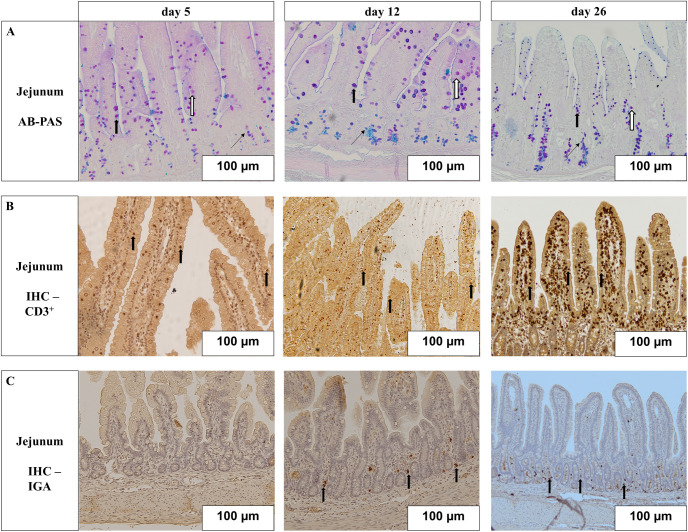
Jejunal histomorphology and immunohistochemistry of 5-, 12-, and 26-day-old suckling piglets. (A) Alcian blue pH 2.5 -periodic acid Schiff stained jejunal tissue with stained goblet cells. Different arrows highlight goblet cells containing different mucins: Narrow arrow = acidic mucins (blue stained), wide closed arrow = neutral mucins (magenta stained, wide open arrow = mixed mucins (purple stained); days 5, 12, 26. (B) Representative immunohistochemistry images of CD3, counterstained with hematoxylin, arrows showing positive stained intraepithelial CD3+ cells in villi; days 5, 12, 26. (C) Representative immunohistochemistry images of IgA positive stained cells in lamina propria. No IgA positive cells were detected at age day 5, arrows indicating IgA positive cells; days 5, 12, 26. Abbreviations: AB-PAS = Alcian blue pH 2.5-periodic acid Schiff, CD 3 = Cluster of differentiation 3, IgA = Immunoglobulin A.

There was an effect of Suppl on VH and the number of mixed mucins containing goblet cells in the villus area (Table 2) (Fig 1), whilst Age affected all other parameters except for the abundance of goblet cells containing acidic mucins in the villus area, and the number of goblet cells containing mixed mucins in the crypt area. At age day 12, VH and the ratio of VH to CD was higher in LBW-Gln compared to LBW-Ala (*P* = 0.031), whilst the number of goblet cells containing neutral, and mixed mucins and the total number of goblet cells in the villus area at age day 26 (*P* = 0.022) was higher in LBW-Ala. At age day 26, the CD was lower in LBW-Gln compared to NBW-Gln (*P* = 0.033). In the 12-day group, VH, the ratio of VH to CD, and the number of goblet cells containing acidic mucins in the crypt area were greater than in the 26-day group (*P* < 0.001). Crypt depth was lower, the total number of goblet cells, as well as the number of goblet cells containing neutral mucins in crypt and villus area, and the number of goblet cells containing mixed mucins in the villus area were lower (*P* < 0.001) in the 12-day than in the 26-day group.

**Table 2:** Acute (12-day group) and persistent (26-day group) effects of an early life (day 1–12) glutamine (Gln) or alanine (Ala) supplementation on jejunal morphometry and the abundance of goblet cells in low (LBW) and normal birthweight (NBW) suckling piglets.

		Ala		Gln					*P-*Value ^*1*^		
Item	Age (day)	LBW	NBW	LBW	NBW	SEM	Age ^2^	SEM	Suppl	BiW	Age
Villus height (μm)	12	899 ^b^	856	1060 ^a^	933	56.1	937 ^A^	33.1	0.017	0.305	<0.001
	26	384	398	441	432	54.1	414 ^B^	31.0			
Crypt depth (μm)	12	149	145	150	143	9.46	147 ^B^	6.28	0.455	0.684	<0.001
	26	254	250	245 ^d^	267 ^c^	8.94	254 ^A^	5.68			
Villus height:crypt depth ratio	12	6.17 ^b^	6.00	7.10 ^a^	6.77	0.428	6.51 ^A^	0.256	0.228	0.067	<0.001
	26	1.58	1.62	1.9	1.67	0.412	1.69 ^B^	0.238			
Villi acidic mucin	12	4.83	4.92	4.68	4.36	0.532	4.70	0.319	0.712	0.865	0.279
	26	5.69	5.13	4.78	4.85	0.512	5.11	0.297			
Villi neutral mucin	12	6.71	7.41	7.12	7.15	0.946	7.10 ^B^	0.513	0.150	0.756	<0.001
	26	19.9 ^a^	17.9	16.9 ^b^	17.1	0.928	17.9 ^A^	0.502			
Villi mixed mucin	12	5.49	6.10	5.12	5.51	0.985	5.55 ^B^	0.509	0.013	0.900	<0.001
	26	22.7 ^a^	20.5	19.3 ^b^	19.6	0.972	20.5 ^A^	0.491			
Villi total mucin	12	17.0	18.4	16.8	16.9	2.09	17.3 ^B^	1.08	0.056	0.851	<0.001
	26	48.2 ^a^	43.5	41.0 ^b^	41.6	2.08	43.6 ^A^	1.06			
Crypt acidic mucin	12	18.2	18.4	19.0	21.9	1.57	19.4 ^A^	0.892	0.089	0.630	<0.001
	26	12.3	12.4	13.0	11.7	1.53	12.3 ^B^	0.844			
Crypt neutral mucin	12	11.7	13.8	12.4	14.2	1.83	13.0 ^B^	0.857	0.870	0.856	<0.001
	26	33.0	33.2	34.3	30.9	1.74	32.8 ^A^	0.805			
Crypt mixed mucin	12	18.3	19.0	17.9	19.3	1.44	18.6	1.18	0.669	0.660	0.052
	26	20.6	20.7	21.6	19.4	1.43	20.6	1.07			
Crypt total mucin	12	48.3	51.2	49.2	55.4	3.31	51.0 ^B^	2.12	0.557	0.888	<0.001
	26	66.1	66.4	68.6 ^c^	61.7 ^d^	3.15	65.7 ^A^	1.93			

Values are LSM ± SEM; 12-day group *N* = 48, *n* = 12/subgroup, 26-day group *N* = 44, *n* = 11/subgroup.

^1^ ANOVA F-Test, none of the interactions (Suppl x BiW; Suppl x Age; BiW x Age or Suppl x BiW x Age) were significant (*P* > 0.05).

^2^ Age; LSM values shown include all values measured within the indicated age group, regardless of BiW and supplementation.

^a,b^ Labeled LSM in a row within one Birthweight group and one Age group without a common letter differ, *P* < 0.05 (Tukey-Kramer test).

^c,d^ Labeled LSM in a row within one Supplementation group and one Age group without a common letter differ, *P* < 0.05 (Tukey-Kramer test).

^A–B^ Indicate significant differences among Age groups *P* < 0.05 (Tukey–Kramer test).

Abbreviations: BiW = Birthweight, Suppl = Supplementation.

### Lymphocyte subsets in jejunal tissue

The factor Age influenced all investigated parameters **(Table 3)**. At age day 26, the number of IgA positive lymphocytes in the villus and crypt area was lower in LBW-Gln compared to NBW-Gln (*P* = 0.004), while the number of intraepithelial CD3 positive lymphocytes was higher in NBW-Gln (*P* = 0.031). The number of IgA and CD3 positive lymphocytes was lower in the 12-day compared to the 26-day group (*P* = 0.008).

**Table 3:** Acute (12-day group) and persistent (26-day group) effects of an early life (day 1–12) glutamine (Gln) or alanine (Ala) supplementation on the abundance of CD3 positive and IgA positive lymphocytes in the jejunum of low (LBW) and normal birthweight (NBW) suckling piglets.

			Ala		Gln					*P-*Value ^*1*^
Item		Age (day)	LBW	NBW	LBW	NBW	SEM	Age ^2^	SEM	Age
IgA positive cells ^3^	Area 1	12	0.333	0.167	0.000	0.083	0.303	0.150 ^B^	0.146	0.007
		26	0.750	0.583	0.167 ^d^	1.42 ^c^	0.303	0.730 ^A^	0.146	
	Area 2	12	0.077	0.327	0.097	0.180	0.481	0.170^B^	0.152	<0.001
		26	1.71	1.29	1.78	1.86	0.468	1.66^A^	0.152	
	Area 3	12	9.84	10.1	8.26	8.93	0.814	9.28^B^	0.268	<0.001
		26	18.3	16.8	16.5 ^d^	18.5 ^c^	0.805	17.5^A^	0.255	
CD3 positive cells ^3^		12	6.61	6.31	6.34	6.75	3.31	6.50^B^	1.53	<0.001
		26	27.1	29.0	28.0 ^d^	30.7 ^c^	3.15	28.7 ^A^	1.34	

Values are LSM ± SEM; 12-day group *N* = 48, *n* = 12/subgroup, 26-day group *N* = 44, *n* = 11/subgroup.

^1^ ANOVA F-Test, none of the other main effects (Suppl. or BiW), or interactions (Suppl x BiW; Suppl x Age; BiW x Age or Suppl x BiW x Age) were significant (*P* > 0.05).

^2^ Age; LSM values shown include all abundances measured within the indicated age group, regardless of BiW and supplementation.

^3^ Group size deviated from *n* = 12 for the parameter CD3+ lymphocytes due to damaging of lamina propria during thawing. NBW-Gln 26-day group, *n* = 10.

^c,d^ Labeled LSM in a row within one Supplementation group and one Age group without a common letter differ, *P* < 0.05 (Tukey-Kramer test).

^A–B^ Indicate significant differences among Age groups *P* < 0.05 (Tukey–Kramer test).

Abbreviations: Area 1 = villus; Area 2 = end of villus/crypt mouth; Area 3 = Area beside the crypt region, BiW = Birthweight, Suppl = Supplementation.

### Incorporation of bromodeoxyuridine in jejunal tissue

Bromodeoxyuridine-positive cells were detected in the crypt (**S1 and S2 Figs)** and in the villus section (**S1 and S3 Figs**). There was a significant effect of Age on all evaluated parameters (**Table 4**). Differences between BiW and Suppl groups were only observed in piglets on age day 26. In this group the ratio of BrdU-positive area to the total area of nuclei was higher in NBW-Gln compared to NBW-Ala (*P* = 0.011), while the same parameter was higher in LBW-Ala compared to NBW-Ala (*P* = 0.021). The percentage of the BrdU-positive area in the crypt section was higher in LBW-Gln compared to NBW-Gln (*P* = 0.009). Total nuclei area in both sections was larger in the 12-day compared to the 26-day group (*P* < 0.001), all other investigated parameters were lower in piglets of the 12-day group (*P* < 0.001).

**Table 4:** Acute (5-day and 12-day groups) and persistent (26-day group) effects of an early life (day 1–12) glutamine (Gln) or alanine (Ala) supplementation on the incorporation of bromodeoxyuridine (BrdU) in jejunal tissue in low (LBW) and normal birthweight (NBW) suckling piglets.

			Ala		Gln					*P*-Value ^*1*^
Item ^2^		Age (day)	LBW	NBW	LBW	NBW	SEM	Age ^3^	SEM	Age
Villus area (%)	Total nuclei area	5	52.0	54.7	58.2	57.7	2.89	55.7 ^A^	52.0	<0.001
		12	58.4	58.6	57.9	59.6	2.94	58.6 ^A^	58.4	
		26	26.9	30.0	30.8	27.9	2.83	28.9 ^B^	26.9	
	BrdU positive nuclei	5	0.620	0.714	0.728	0.786	0.340	0.712 ^B^	0.225	<0.001
		12	0.762	0.678	0.66	0.896	0.331	0.749 ^B^	0.224	
		26	3.73	3.23	4.08	4.17	0.325	3.80 ^A^	0.204	
	Ratio BrdU positive area to Total nuclei area	5	0.014	0.014	0.013	0.014	0.014	0.014 ^B^	0.014	<0.001
		12	0.014	0.013	0.012	0.015	0.013	0.014 ^B^	0.014	
		26	0.151 ^c^	0.108 ^d, b^	0.142	0.156 ^a^	0.013	0.142 ^A^	0.151	
Crypt area (%)	Total nuclei area	5	61.7	60.6	65.8	63.9	2.27	63.2 ^A^	1.66	<0.001
		12	58.3	58.9	58.0	59.5	2.22	58.7 ^A^	1.68	
		26	37.5	38.7	40.3	36.5	2.15	38.2 ^B^	1.49	
	BrdU positive nuclei	5	15.7	15.0	15.2	14.7	1.27	15.1 ^B^	0.901	<0.001
		12	14.4	13.6	14.5	15.1	1.26	14.4 ^B^	0.909	
		26	20.4	18.9	20.5 ^c^	17.1 ^d^	1.18	19.2 ^A^	0.809	
	Ratio BrdU positive area to Total nuclei area	5	0.257	0.236	0.231	0.228	0.029	0.24 ^B^	0.019	<0.001
		12	0.248	0.238	0.250	0.252	0.028	0.25 ^B^	0.019	
		26	0.562	0.500	0.522	0.466	0.028	0.51 ^A^	0.017	

Values are LSM ± SEM; 5-day group *N =* 45, 12-day group *N* = 48, *n* = 12/subgroup, 26-day group *N* = 44, *n* = 11/subgroup.

^1^ANOVA F-Test, none of the other main effects (Suppl. or BiW), or interactions (Suppl x BiW; Suppl x Age; BiW x Age or Suppl x BiW x Age) were significant (*P* > 0.05).

^2^Group size deviated from *n* = 12 for bromodeoxyuridine related parameters because of insufficient accumulation of bromodeoxyuridine in jejunal tissue of 5-day groups NBW-Gln, NBW-Ala, LBW-Gln *n* = 11; LBW-Ala *n* = 12.

^3^Age, LSM values shown include all values measured within the respective age group, regardless of BiW and supplementation.

^a,b^ Labeled LSM in a row within one Birthweight group and one Age group without a common letter differ, *P* < 0.05 (Tukey-Kramer test).

^c,d^ Labeled LSM in a row within one Supplementation group and one Age group without a common letter differ, *P* < 0.05 (Tukey-Kramer test).

^A–B^ Indicate significant differences among Age groups *P* ≤ 0.05 (Tukey–Kramer test).

Abbreviations: BiW = Birthweight, BrdU = bromodeoxyuridine, Suppl = Supplementation.

#### Transcript abundance and location of tight junction proteins in jejunal tissue.

There was an effect of BiW on the transcript abundance of TJP1, CLDN4, and OCLN (Table 5). At age day 26, the mRNA abundance of TJP2 was higher in NBW-Gln compared to NBW-Ala (*P* = 0.019), and the abundance of TJP2 was higher in LBW-Ala compared to NBW-Ala (*P* = 0.003). At age day 5, TJP1 and OCLN abundance was higher in LBW-Ala compared to NBW-Ala (*P* = 0.011). In all BiW and Age groups, TJP1 (**S4** and **S5 Figs**) and TJP2 (**S6** and **S7 Figs**) were located at the basolateral and the apical side of intestinal epithelial cells. Also, CLDN4 (**S8** and **S9 Figs**) and OCLN (**S10** and **S11 Figs)** were detectable in crypt as well as in the villus region and were identified at the basolateral and apical side of intestinal epithelial cells. There were no obvious differences in the location of these TJP depending on the main factors assessed.

**Table 5:** Acute (5-day and 12-day groups) and persistent (26-day group) effects of an early life (day 1–12) glutamine (Gln) or alanine (Ala) supplementation on the mRNA abundance of tight junction proteins, and targets of cellular proliferation and apoptosis in low (LBW) and normal birthweight (NBW) suckling piglets.

		Ala		Gln					*P-Value* ^*1*^
Item ^2^	Age (day)	LBW	NBW	LBW	NBW	SEM	Age ^3^	SEM	BiW
TJP1	5	0.231 ^c^	-0.077 ^d^	0.047	0.110	0.082	0.078	0.041	0.017
	12	-0.028	-0.027	0.008	-0.015	0.082	-0.015	0.041	
	26	0.044	-0.009	0.017	-0.032	0.085	0.005	0.043	
TJP2	5	0.171	0.151	0.121	-0.069	0.092	0.094	0.049	0.031
	12	-0.061	-0.047	0.040	0.012	0.092	-0.014	0.050	
	26	0.161 ^c^	-0.248 ^b^^,^ ^d^	0.106	0.063 ^a^	0.095	0.021	0.050	
CLDN4	5	0.195	0.138	0.033	0.003	0.081	0.092	0.044	0.114
	12	-0.009	-0.091	0.014	0.031	0.082	-0.014	0.045	
	26	0.072	-0.155	0.084	0.031	0.084	0.008	0.045	
OCLN	5	0.254 ^c^	-0.079 ^d^	0.127	-0.060	0.098	0.061	0.053	0.012
	12	-0.046	-0.045	-0.001	0.016	0.097	-0.019	0.053	
	26	0.025	-0.174	-0.025	-0.157	0.101	-0.083	0.054	
PCNA	5	0.058	0.000	0.077	0.115	0.065	0.063	0.033	0.520
	12	-0.043	0.056	-0.020	0.026	0.065	0.004	0.033	
	26	0.005	0.049	-0.029	-0.049	0.068	-0.006	0.034	
CASP3	5	0.018	-0.077	0.025	0.022	0.092	-0.003	0.056	0.490
	12	-0.037	0.030	-0.014	-0.025	0.094	-0.011	0.058	
	26	-0.015	-0.112	-0.001	-0.054	0.094	-0.045	0.056	

Values are LSM ± SEM; 5-day group *N* = 48, *n* = 12/subgroup, 12-day group *N* = 48, *n* = 12/subgroup, 26-day group *N* = 44, *n* = 11/subgroup.

^1^ ANOVA F-Test, none of the other main effects (Suppl. or Age), or interactions (Suppl x BiW; Suppl x Age; BiW x Age or Suppl x BiW x Age) were significant (*P* > 0.05).

^2^ mRNA abundance (log-CNRQ).

^3^ Age; LSM values shown include all values measured within the respective age group, regardless of BiW and supplementation.

^a,b^ Labeled LSM in a row within one Birthweight group and one Age group without a common letter differ, *P* < 0.05 (Tukey-Kramer test).

^c,d^ Labeled LSM in a row within one Supplementation group and one Age group without a common letter differ, *P* < 0.05 (Tukey-Kramer test).

Abbreviations: BiW = Birthweight, CASP 3 = Caspase 3, OCLN = Occludin, PCNA = Proliferating-Cell-Nuclear-Antigen, Suppl = Supplementation, TJP1 = Tight junction protein 1, TJP2 = Tight junction protein 2.

### Amino acid and glutathione concentration in plasma and jejunal tissue

#### Free amino acid concentration in plasma.

There was an effect of Suppl on the plasma concentration of Ala, Arg, Gln and Pro, while BiW affected Orn and Pro concentration. Age influenced the concentration of all evaluated AAs (**Table 6**). At age day 12, the plasma Ala concentration was higher in Ala supplemented compared to Gln supplemented piglets (*P* < 0.001), the concentration of Arg and Gln was higher in NBW-Gln compared to NBW-Ala (*P* = 0.023*)*, and the concentration of Ala was higher in LBW-Ala than in NBW-Ala (*P* = 0.001). The concentration of all investigated AAs as well as the concentration of the non-proteinogenic citrulline and Orn was higher in the 12-day group (*P* < 0.001), whereas the concentration of Glu was higher in 26-day group (*P* < 0.001).

**Table 6:** Acute (12-day group) and persistent (26-day group) effects of an early life (day 1–12) glutamine (Gln) or alanine (Ala) supplementation on the concentration of free and protein-bound amino acids (FAA, PBAA), in plasma and jejunal tissue as well as the concentration of glutathione in jejunal tissue in low (LBW) and normal birthweight (NBW) suckling piglets.

			Ala		Gln					*P-*Value ^1^			
Item		Age (day)	LBW	NBW	LBW	NBW	SEM	Age ^2^	SEM	Suppl	BiW	Age	Interaction ^3^
Plasma FAA (μmol/L)	Ala	12	1297 ^a^^,^ ^c^	1093 ^a, d^	652 ^b^	705 ^b^	86.7	937 ^A^	54.6	< 0.001	0.793	< 0.001	0.230
		26	731	742	733	820	86.3	756 ^B^	52.6				
	Arg	12	183	155 ^b^	190.5	200 ^a^	13.0	182 ^A^	7.74	0.034	0.942	0.001	0.490
		26	99.7	98.9	90.6	87.3	13.1	94.1 ^B^	7.58				
	Cit	12	119	119	132	129	9.13	124 ^A^	6.21	0.374	0.395	< 0.001	0.689
		26	83.6	76.9	76.8	70.9	8.97	77.0 ^B^	5.88				
	Gln	12	522	536 ^b^	627	681 ^a^	50.3	592 ^A^	32.1	< 0.001	0.716	< 0.001	0.714
		26	552	544	507	587	50.0	547 ^B^	30.8				
	Glu	12	218	198	218	221	14.9	214 ^B^	21.3	0.213	0.267	< 0.001	0.771
		26	342	342	353	373	14.1	353 ^A^	20.9				
	Orn	12	94.9	83.2	98.0	88.1	7.46	91.1 ^A^	4.96	0.983	0.041	< 0.001	0.675
		26	63.4	55.5	55.5	54.7	7.36	57.3 ^B^	4.72				
	Pro	12	863	747	742	703	123	764 ^A^	85.4	0.042	0.111	< 0.001	0.667
		26	566	455	469	557	121	512 ^B^	80.6				
Jejunum FAA (μmol/g FM)	Ala	12	3.89	4.17	4.11	3.71	0.41	3.97 ^A^	0.241	0.736	0.595	< 0.001	0.056
		26	2.64	3.13	2.96	2.80	0.41	2.88 ^B^	0.236				
	Arg	12	1.61	1.79	2.02	1.86	0.255	1.82 ^A^	0.162	0.362	0.738	< 0.001	0.071
		26	0.496	0.718	0.609	0.567	0.254	0.598 ^B^	0.156				
	Cit	12	12	0.192	0.207	0.230	0.201	0.022	0.207	0.893	0.189	0.890	0.467
		26	26	0.203	0.238	0.187	0.183	0.022	0.203				
	Gln	12	1.60	1.83	2.13	1.95	0.229	1.87 ^A^	0.15	0.736	0.015	< 0.001	0.041
		26	0.954	1.10	1.01	1.05	0.226	1.03 ^B^	0.14				
	Glu	12	6.69	7.29	7.35	6.69	0.538	7.00 ^B^	0.309	0.015	0.898	<0.001	0.405
		26	8.50	8.68	8.47	9.21	0.545	8.72 ^A^	0.306				
	Orn	12	0.079	0.079	0.075	0.063	0.013	0.074	0.007	0.683	0.439	0.812	0.532
		26	0.082	0.074	0.080	0.049	0.014	0.071	0.007				
	Pro	12	2.47	2.82	3.34	2.91	0.53	2.89 ^A^	0.31	0.683	0.439	0.812	0.095
		26	1.41	1.87	1.91	1.79	0.54	1.74 ^B^	0.30				
Jejunum PBAA (μmol/g FM)	Ala	12	42.4	42.7	44.7	40.7	13.4	42.6	7.20	0.660	0.196	< 0.001	0.973
		26	47.0	48.9	49.3	48.6	13.8	48.4	7.30				
	Arg	12	29.9	30.2	31.0	28.3	8.75	29.9	4.78	0.660	0.196	< 0.001	0.924
		26	33.2	33.5	33.8	33.4	8.95	33.2	4.81				
	Gln	12	24.4	24.5	25.6	23.4	6.93	24.5	3.72	0.325	0.181	< 0.001	0.894
		26	26.8	28.0	27.9	27.8	7.11	27.6	3.76				
	Glu	12	38.6	39.6	41.6	38.1	12.9	39.5	6.56	0.392	0.200	< 0.001	0.927
		26	43.2	45.6	45.7	44.7	13.4	44.8	6.56				
	Pro	12	27.6	23.7	26.4	23.9	17.6	25.4	10.2	0.075	0.544	< 0.001	0.831
		26	25.4	25.6	27.6	27.0	17.8	26.4	10.1				
Jejunum Total Protein (g/g FM)		12	0.084	0.083	0.087	0.084	0.003	0.084 ^B^	0.001	0.744	0.721	< 0.001	0.015
		26	0.111 ^a^	0.110	0.106 ^b,c^	0.114 ^d^	0.003	0.111 ^A^	0.001				
Jejunum Glutathione (μmol/ g Protein)		12	15.5 ^c^	12.8 ^d^	13.7	13.9	1.10	14.0^A^	0.662	0.585	0.046	0.010	0.208
		26	11.5	10.3	12.9	11.2	1.05	11.5^B^	0.627				

Values are LSM ± SEM; 12-day group *N* = 48, *n* = 12/subgroup, 26-day group *N* = 44, *n* = 11/subgroup.

^a,b^ Labeled LSM in a row within one Birthweight group and one Age-group without a common letter differ, *P* < 0.05 (Tukey-Kramer test).

^c,d^ Labeled LSM in a row within one Supplementation group and one Age-group without a common letter differ, P < 0.05 (Tukey-Kramer test).

^A–B^ indicate significant differences among Age groups *P* ≤ 0.05 (Tukey–Kramer test).

^1^ ANOVA F-Test, none of the other interactions (Suppl x BiW, Suppl x Age, BiW x Age) were significant (*P* > 0.05).

^2^ Age; LSM values shown include all values measured within the respective Age group, regardless of BiW and supplementation.

^3^ Interaction Suppl x BiW x Age.

Abbreviations: BiW = Birthweight, Cit = Citrulline, FM = Fresh matter, Orn = Ornithine Suppl = Supplementation.

#### Free amino acid and amino acid metabolite concentration in jejunal tissue.

In jejunal tissue, the concentration of Gln was affected by BiW, whereas Age affected all other parameters with exception of citrulline, Pro, and Orn. The interaction of Suppl x BiW x Age affected Gln concentration. The concentration of Ala, Arg, Gln and Pro was higher in the 12-day group (*P* < 0.001), while the concentration of Glu was higher in the 26-day group (*P* < 0.001) (Table 6).

#### Protein bound amino acid and glutathione concentration in jejunal tissue.

The factor Age affected all measured parameters, whereas the interaction Suppl x BiW x Age influenced total protein concentration. On age day 26 the total protein concentration was lower in LBW-Gln than LBW-Ala (*P* = 0.026), whereas total protein concentration was lower in LBW-Gln than NBW-Gln (*P* = 0.024) (Table 6).

The factors BiW and Age influenced the concentration of glutathione. On age day 12 the concentration of glutathione was higher in LBW-Ala than in NBW-Ala (*P* = 0.042). The total GSH concentration was higher in the 12-day group than in 26-day group (*P* = 0.014).

## Discussion

There is limited research on the effects of pre‐weaning nutritional support on development and gut health prior to weaning in piglets [1]. We have previously shown that the supplementation of Gln from age day 1 to 12 increased growth and milk intake in LBW piglets which was associated to changes in lipid metabolism suggesting Gln effects on the splanchnic tissue [31]. Additionally, in a companion paper we reported that Gln supplementation resulted in Gln accumulation in skeletal muscle of 5-day old piglets and that Gln supplementation was associated with larger muscle fibers [42], as well as higher cellular proliferation in skeletal muscle [43]. Collectively, these results appear to support earlier findings on the growth-promoting effect of glutamine in pre-weaning piglets [21,22]. Therefore, the aims of this study were (1) to explore whether the growth advantage of Gln-supplemented LBW piglets during the acute phase of supplementation (age day 1 to 12) persists as “carry-over-effect” after supplementation has ended, and (2) if this was associated with changes in jejunal development and maturation.

### Comparison among supplementation groups

In the current study, LBW piglets supplemented with Gln were heavier than their Ala supplemented littermates until age day 21, 9 days after the cessation of supplementation. Analysis of jejunal samples collected on the final supplementation day (12) and two weeks after cessation of supplementation (26 days), showed only minimal effects of Gln supplementation on jejunal morphology, cellular populations, tissue AA and glutathione concentrations, consistent with results previously published by our group [32], investigating piglets during the supplementation period (age days 5 and 12). Additionally, only minor effects of Gln supplementation on jejunal cellular proliferation and tight junction protein mRNA abundance were observed in piglets during (5 and 12 days) and after cessation of supplementation (26 day). Except for increased VH and ratio of VH to CD in 12-day old LBW piglets supplemented with Gln compared to Ala littermates, none of the observed differences could explain the improved growth performance of LBW piglets supplemented with Gln. We have previously discussed this in detail [32], focusing on two main hypotheses. Since Gln supplementation did not increase jejunal digesta Gln concentrations [32], we assumed that Gln was absorbed, as indicated by the increased concentration of free Gln and related AA in the jejunum tissue of 12- vs. 26-day group piglets, but might have affected intestinal development in more proximal sections of the SI. Alternatively, Gln may exert its beneficial effects on the SI rather in stressed or diseased animals [44]. It seems that low birthweight itself, even below the lowest quartile birthweight category and with lower survival probability [33,34], does not compromise SI function and development to the extent that Gln could have a beneficial effect.

The increase in VH and VH to CD ratio observed in this study might be an indicator for an increase of mucosal surface [24]. Morphologic measurements have been used as markers of absorptive capacity [45] and it has been shown that Gln supplementation improved absorptive capacity in weaned piglets [46]. Improved absorptive capacity could be a transient advantage for the growth of LBW piglets supplemented with Gln compared to those supplemented with Ala. However, it cannot be excluded that improved growth was also associated to a higher milk intake observed from day 11 to 12 shortly before cessation of Gln supplementation as reported [31]. In contrast, in the current study we found that at age day 26 LBW piglets supplemented with Gln consumed equal amounts of milk as LBW supplemented with Ala, suggesting that there is no sustained effect of Gln on milk intake. This may explain why the BW of LBW piglets supplemented with Gln was no longer higher than that of Ala supplemented LBW littermates at the age of day 26. In another study Gln supplementation was not associated with differences in milk intake of suckling piglets [22]. In addition, energy, and nutrient intake at age day 26 did not come exclusively from milk, because piglets had access to solid feed between age 14 to 26 days, which may have contributed to better piglet growth [1] but could not be quantified in the present study. Indeed individual creep feed intake could have also explained the observed differences in TJP mRNA abundance, cell proliferation and goblet cell abundance at age day 26. It was shown that individual creep feed intake in suckling piglets was negatively correlated with the number of PCNA-positive cells and the ratio of VH to CD in jejunal tissue [47], indicating that differences in individual creep feed intake can affect jejunal development irrespective of glutamine supplementation. However, in another study the supplementation of glutamine-enriched creep feed (1%) pre-weaning (age 14–21 days) and of feed during 3 weeks post-weaning improved the feed conversion ratio compared to controls (standard creep feed) but did not affect the litter-based feed intake [46].

It has been shown that glutamine increased the rate of cell proliferation in porcine intestinal cell culture as well as TJP transcript abundance and protein levels, while it decreased paracellular permeability (IPEC cells) [7,48]. However, the results of our *in vivo* studies do not support these findings. We also analyzed fractional protein synthesis rate in the jejunum of the same piglets but likewise did not observe an effect of glutamine on this parameter as reported earlier [32]. This is in contrast to reports showing beneficial effects of glutamine on fractional protein synthesis rate in porcine intestinal cell culture [49]. Presumably, the reason for the discrepancies between *in vitro* glutamine supplementation studies, and our *in vivo* study is the concentration of glutamine. In the cell cultures, glutamine concentrations were 2–8 times higher than the plasma glutamine concentrations in our Gln supplemented piglets [31]. Therefore, translation of *in vitro* to *in vivo* studies examining the effects of glutamine is difficult because it is assumed that effects found *in vitro* can be reproduced *in vivo*, although the systemic conditions are fundamentally different.

### Comparison among birthweight groups

Throughout the study, LBW piglets from both supplementation groups remained smaller and lighter than their NBW littermates, whilst small differences in jejunal morphology, cellular population, and proliferation, TJP transcript abundance and glutathione concentration were observed between LBW and NBW piglets. However, these differences did not allow conclusions regarding systematic developmental differences between LBW and NBW piglets in the jejunum. Other studies in piglets of similar age to the current study showed that LBW was not associated with impairment of SI morphology, cellular proliferation, or goblet cell abundance [50,51] This observation is confirmed by our study. However, there is conflicting evidence on whether BiW affects jejunal barrier function [51,52]. In this study we detected an effect of BiW on the transcript abundances of TJP1, TJP2 and OCLN. However, impaired barrier function has been associated with higher [53] and lower TJP transcript abundance [52]. Both higher and lower TJP transcript abundance has been linked to elevated interleukin levels [53] or higher oxidative stress [52]. In our experiment altered TJP mRNA levels were not associated with lower glutathione concentrations in jejunal tissue, changes in TJP location or increased immigration of inflammatory cells. There is also conflicting evidence whether LBW impairs glutathione metabolism in jejunal tissue during the suckling period [32,52]. Low birthweight has been associated with lower abundance of intraepithelial lymphocytes [54], however this could not be confirmed in the current study. This might be due to the fact that in the study of Prims et al. 2016 [54] other subsets of intraepithelial lymphocytes (CD8+ and CD4+ lymphocytes) were determined. Different subsets of lymphocytes fulfill diverging functions in the mucosal immunity and a pan intraepithelial lymphocyte marker has not been established [55]. Thus to assess how a low birthweight affects the development of jejunal mucosal immunity is challenging because different subpopulations of intraepithelial lymphocytes with different functions are compared. Together the results of the current study suggest that LBW piglets that survive the first days after birth do not have impaired intestinal development if they consume sufficient amounts of colostrum [24].

### Comparison among age groups

The factor Age showed the clearest effect on the measured intestinal parameters among all factors analyzed in this study. In previous studies parameters of small intestinal development such as morphology [55], cell populations or barrier function [16] differ between the first and the fourth week of life in suckling pigs. It has been known for some time that jejunal cellular proliferation is higher in the fourth week of life than in the first and has been associated with a lower susceptibility of older suckling piglets to enteral viral infections [56]. Results from this study confirm that cellular proliferation increases during the suckling period, regardless of birthweight.

Piglets are born without an acquired immune system and an incomplete mucosal immunity therefore rely on immunoglobulin intake from maternal colostrum [57]. Under commercial conditions, piglets are weaned with an incompletely adapted enteric immune system [1,57]. Our IHC-analysis shows that the number of intraepithelial lymphocytes was higher in the 26-day group than in the 12-day group but the number of intraepithelial lymphocytes is still lower than in adult pigs, suggesting that the development of the intestinal mucosal is not yet complete [58]. A similar porcine developmental pattern has also been described by others [59]. In addition, intraepithelial lymphocytes have been shown to migrate into the jejunal mucosa after birth and this process is also regulated by the uptake of colostral bioactive macromolecules [58]. Additional factors have been identified that influence jejunal development, including ontogenetic factors, dietary changes and microbiome composition [16]. As sow milk yield peaks in the third week of lactation and becomes limiting thereafter [60], additional creep feed is commonly offered to suckling piglets preparing them for weaning and supporting their growth [1]. There is evidence that creep feed might influence jejunal morphometry independent of the jejunal microbial population [47]. In contrast, jejunal mucosal development was shown to be correlated negatively with the *Enterobacteriaceae* population in the jejunum [16], while the effect of age on total microbial population was less distinct in jejunum than in colon. A companion study using the same animals as in this report and examining developmental parameters of the colon, including mucosa architecture and colon microbiota, also showed that age had the greatest effect among all influencing factors examined [61]. Based on current knowledge, the exact mechanisms of jejunal development are still unclear and warrant further research. The observed differences in jejunal mucosal morphology, cell population and proliferation might therefore be related to the influence of a combination of ontogenetic factors, dietary changes and possibly microbial factors.

## Conclusion

This study showed that oral Gln supplementation between age days 1 to 12 improved the BW of LBW piglets compared to Ala-supplemented LBW littermates from day 12 to day 21 of age. However, Gln had a very limited effect on parameters of morphology, TJP transcript abundance, and oxidative status in jejunal tissue. We found little evidence that low BiW affected the measured jejunal parameters under the current experimental conditions. As other studies have shown, the jejunum develops rapidly during suckling, suggesting that ontogenetic factors have a more significant influence on intestinal development than BiW and Gln supplementation. This study provides further evidence that neonatal Gln supplementation can improve the growth of pre-weaning LBW piglets, and additional studies should be conducted to understand the metabolic fate of Gln in suckling piglets underpinning this observation.

## Supporting information

S1 Fig Bromodeoxyuridine incorporation into proliferating jejunal cells. The upper pictures represent propidium iodide stained nuclei (red florescent; left) and bromodeoxyuridine incorporating nuclei (green florescent; right) in the section of the crypt area (100 x magnification). The lower pictures represent propidium iodide stained nuclei (red florescent; left) and bromodeoxyuridine incorporating nuclei (green florescent; right) in the section of the villus area (100x magnification).
(TIF)

S2 Fig Bromodeoxyuridine incorporation in the crypt area of jejunal cross sections. Bromodeoxyuridine incorporating nuclei (green florescent) in the crypt area of jejunal cross sections (100 x magnification) of Glutamine (Gln) and Alanine (Ala) supplemented low (LBW) and normal birthweight (NBW) suckling piglets in three different age groups (5, 12, 26 days).
(TIF)

S3 Fig Bromodeoxyuridine incorporation in proliferating jejunal cells of the villus area. Bromodeoxyuridine incorporating nuclei (green florescent) in the villus area of jejunal cross sections (100 x magnification) of Glutamine (Gln) and Alanine (Ala) supplemented low (LBW) and normal birthweight (NBW) suckling piglets in three different age groups (5, 12, 26 days).
(TIF)

S4 Fig Immunohistochemical images of tight junction protein 1 in the villus area of jejunal cross sections. Immunohistochemical images of tight junction protein 1 (green) in low and normal birthweight piglets in three different age groups (5, 12, 26 days); nuclei were counterstained with Hoechst 33258 (blue), scale bars represent 100 μm.
(TIF)

S5 Fig Immunohistochemical images of tight junction protein 1 in the crypt area of jejunal cross sections. Immunohistochemical images of tight junction protein 1 (green) in low and normal birthweight piglets in three different age groups (5, 12, 26 days); nuclei were counterstained with Hoechst 33258 (blue), scale bars represent 100 μm.
(TIF)

S6 Fig Immunohistochemical images of tight junction protein 2 in the villus area of jejunal cross sections. Immunohistochemical images of tight junction protein 2 (green) in low and normal birthweight piglets in three different age groups (5, 12, 26 days); nuclei were counterstained with Hoechst 33258 (blue), scale bars represent 100 μm.
(TIF)

S7 Fig Immunohistochemical images of tight junction protein 2 in the crypt area of jejunal cross sections. Immunohistochemical images of tight junction protein 2 (green) in low and normal birthweight piglets in three different age groups (5, 12, 26 days); nuclei were counterstained with Hoechst 33258 (blue), scale bars represent 100 μm.
(TIF)

S8 Fig Immunohistochemical images of claudin-4 in in the villus area of jejunal cross sections. Immunohistochemical images of claudin-4 (red) in low and normal birthweight piglets in three different age groups (5, 12, 26 days); nuclei were counterstained with Hoechst 33258, scale bars represent 100 μm.
(TIF)

S9 Fig Immunohistochemical images of claudin-4 in the crypt area of jejunal cross sections. Immunohistochemical images of claudin-4 (red) in low and normal birthweight piglets in three different age groups (5, 12, 26 days); nuclei were counterstained with Hoechst 33258, scale bars represent 100 μm.
(TIF)

S10 Fig Immunohistochemical images of occludin in the villus area of jejunal cross sections. Immunohistochemical images of occludin (red) in low and normal birthweight piglets in three different age groups (5, 12, 26 days); nuclei were counterstained with Hoechst 33258 (blue), scale bars represent 100 μm.
(TIF)

S11 Fig Immunohistochemical images of occludin in the crypt area of jejunal cross sections. Immunohistochemical images of occludin (red) in low and normal birthweight piglets in three different age groups (5, 12, 26 days); nuclei were counterstained with Hoechst 33258 (blue), scale bars represent 100 μm.
(TIF)

S1 Table Nutrient composition of supplementary creep feed. (DOCX)

S2 Table Primer sequences. (DOCX)

## Abbreviations:

AA: Amino acid
AC: Abdominal circumference
Age: age group
Ala: Alanine
BiW: Birthweight
BMI: Body mass index
BrdU: Bromodeoxyuridine
CD: Crypt depth
CD3: Cluster of differentiation 3
CLDN4: Claudin-4
CRL: Crown-rump length
D_2_O: Deuterium oxide
Gln: Glutamine
IgA: Immunoglobulin A
LBW: Low birthweight
LSM: Least square mean
NBW: Normal birthweight
OCLN: Occludin
Orn: Ornithine
PBS: Phosphate-buffered saline
PBST: 0.1% Triton X-100 in phosphate-buffered saline
SEM: Standard error of the mean
SI: Small intestine
Suppl: Supplementation
TJP: Tight junction protein
TJP1: Tight junction protein 1
TJP2: Tight junction protein 2
VH: Villus height
