# Supplementary figures and images for: Acute and persistent effects of oral glutamine supplementation on growth, cellular proliferation, and tight junction protein transcript abundance in jejunal tissue of low and normal birthweight pre-weaning piglets
Source: PLoS One. 2024 Jan 2;19(1):e0296427. doi: 10.1371/journal.pone.0296427 (PMC10760696; doi:10.1371/journal.pone.0296427)

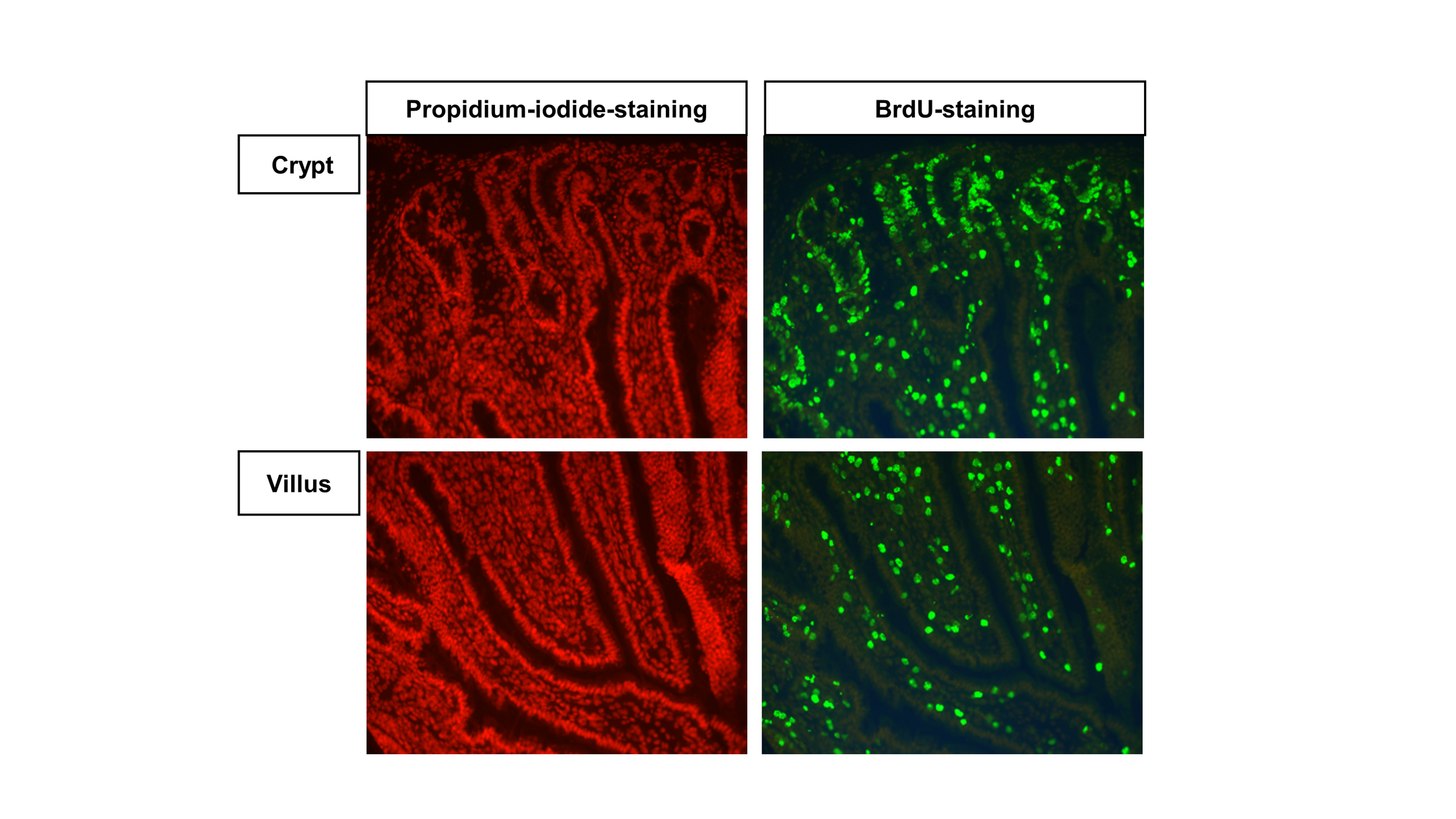

Supplement: S1 Fig — The upper pictures represent propidium iodide stained nuclei (red florescent; left) and bromodeoxyuridine incorporating nuclei (green florescent; right) in the section of the crypt area (100 x magnification). The lower pictures represent propidium iodide stained nuclei (red florescent; left) and bromodeoxyuridine incorporating nuclei (green florescent; right) in the section of the villus area (100x magnification). (TIF) [file pone.0296427.s001.tif]

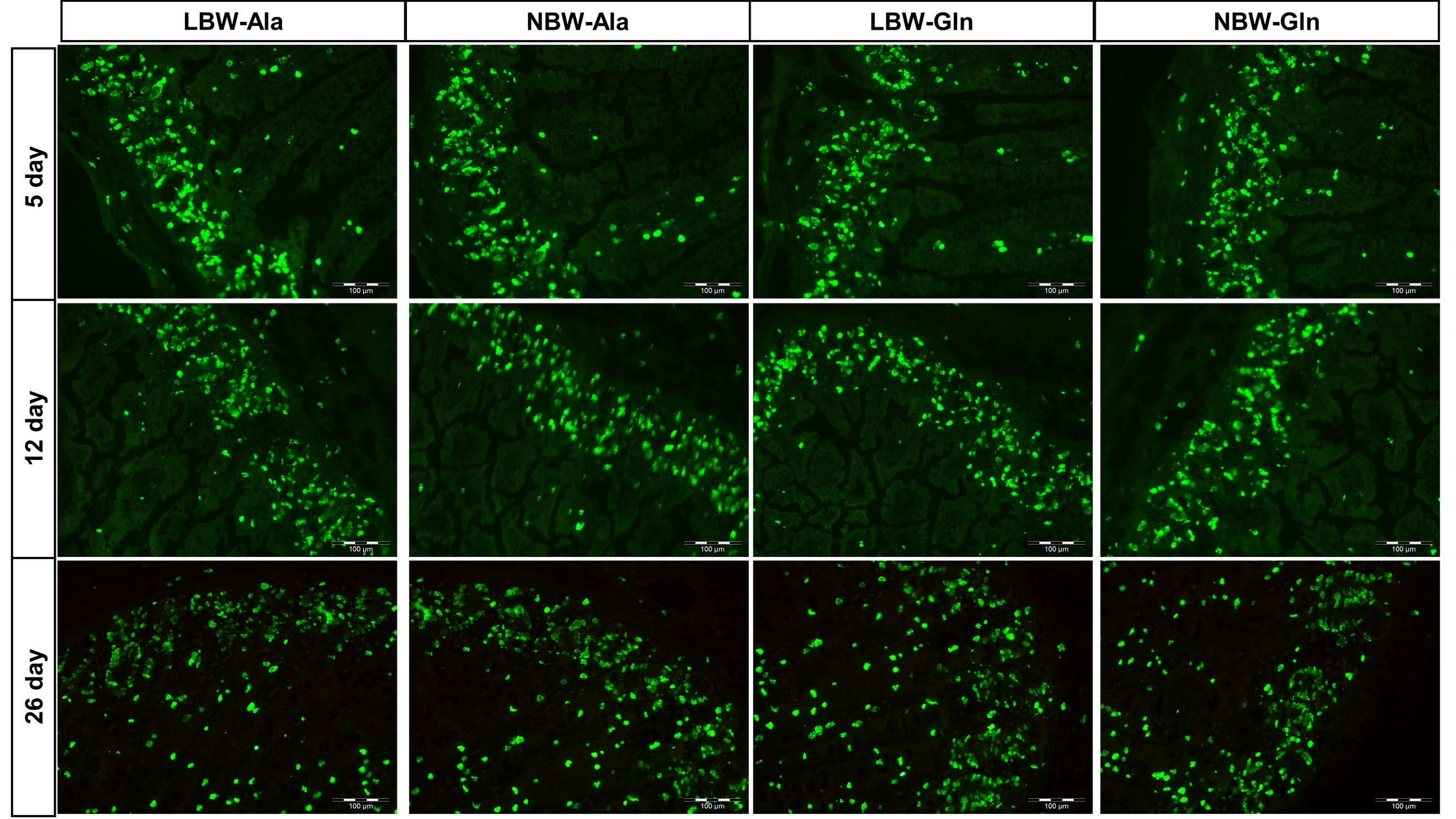

Supplement: S2 Fig — Bromodeoxyuridine incorporating nuclei (green florescent) in the crypt area of jejunal cross sections (100 x magnification) of Glutamine (Gln) and Alanine (Ala) supplemented low (LBW) and normal birthweight (NBW) suckling piglets in three different age groups (5, 12, 26 days). (TIF) [file pone.0296427.s002.tif]

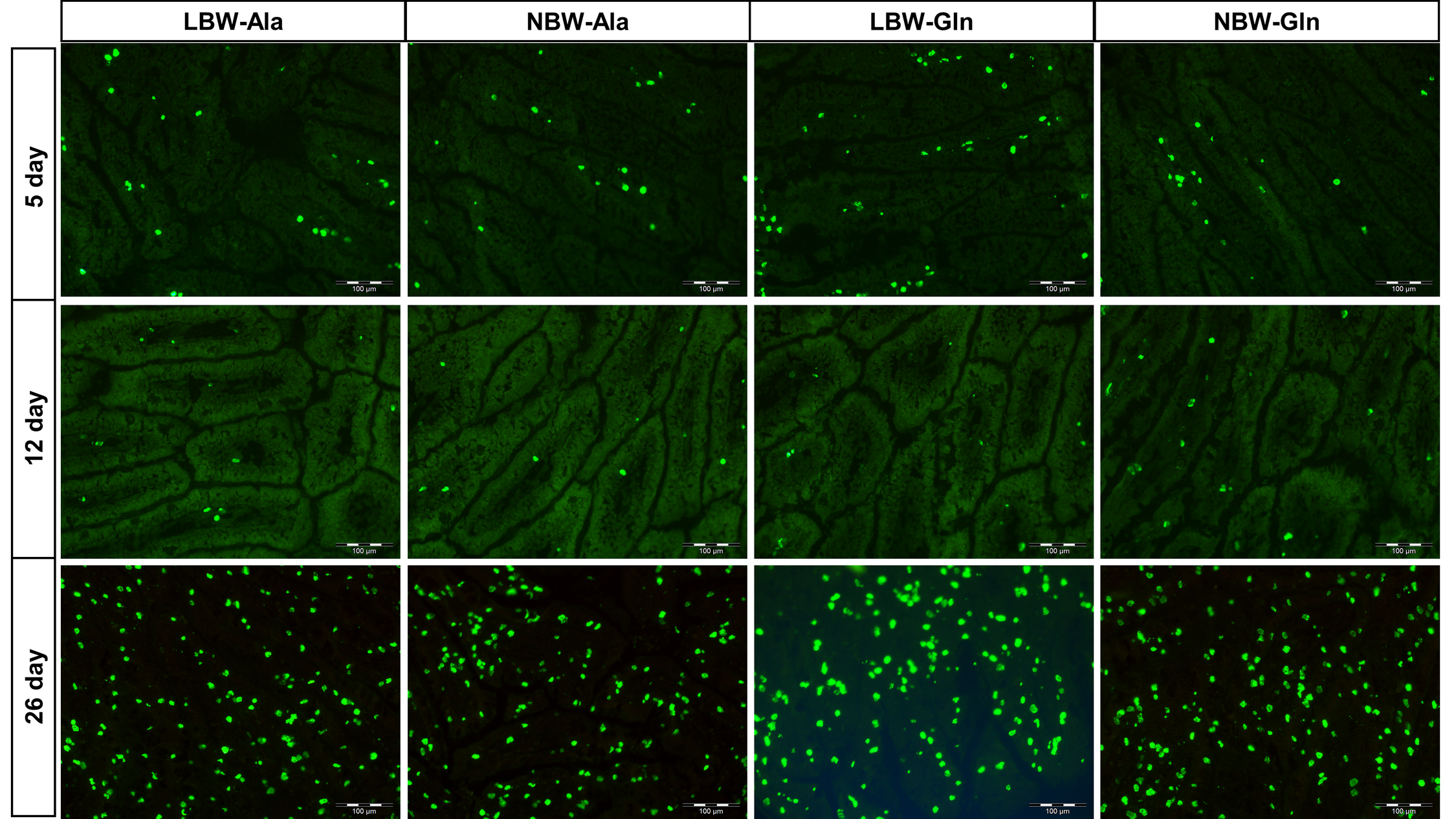

Supplement: S3 Fig — Bromodeoxyuridine incorporating nuclei (green florescent) in the villus area of jejunal cross sections (100 x magnification) of Glutamine (Gln) and Alanine (Ala) supplemented low (LBW) and normal birthweight (NBW) suckling piglets in three different age groups (5, 12, 26 days). (TIF) [file pone.0296427.s003.tif]

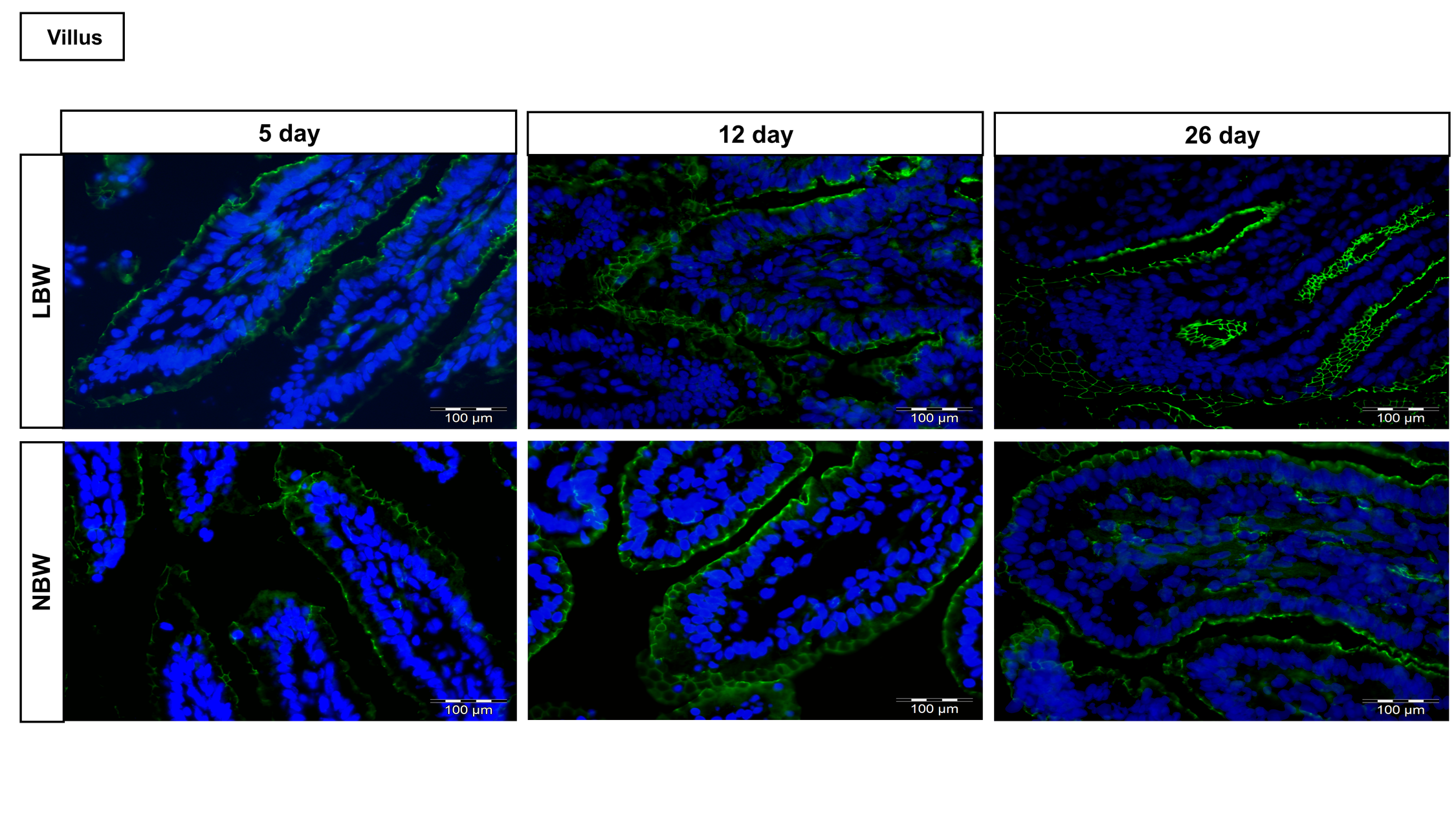

Supplement: S4 Fig — Immunohistochemical images of tight junction protein 1 (green) in low and normal birthweight piglets in three different age groups (5, 12, 26 days); nuclei were counterstained with Hoechst 33258 (blue), scale bars represent 100 μm. (TIF) [file pone.0296427.s004.tif]

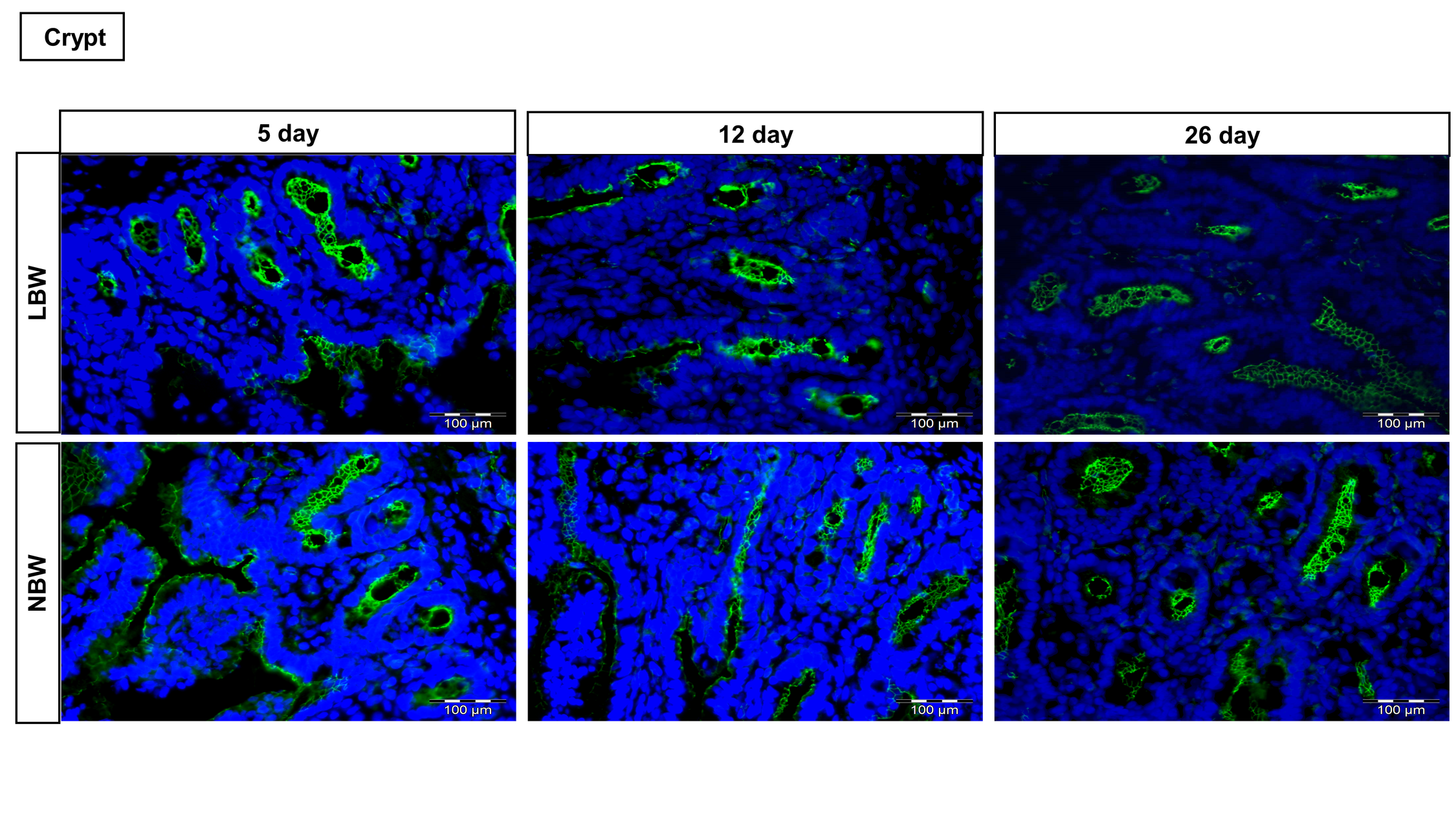

Supplement: S5 Fig — Immunohistochemical images of tight junction protein 1 (green) in low and normal birthweight piglets in three different age groups (5, 12, 26 days); nuclei were counterstained with Hoechst 33258 (blue), scale bars represent 100 μm. (TIF) [file pone.0296427.s005.tif]

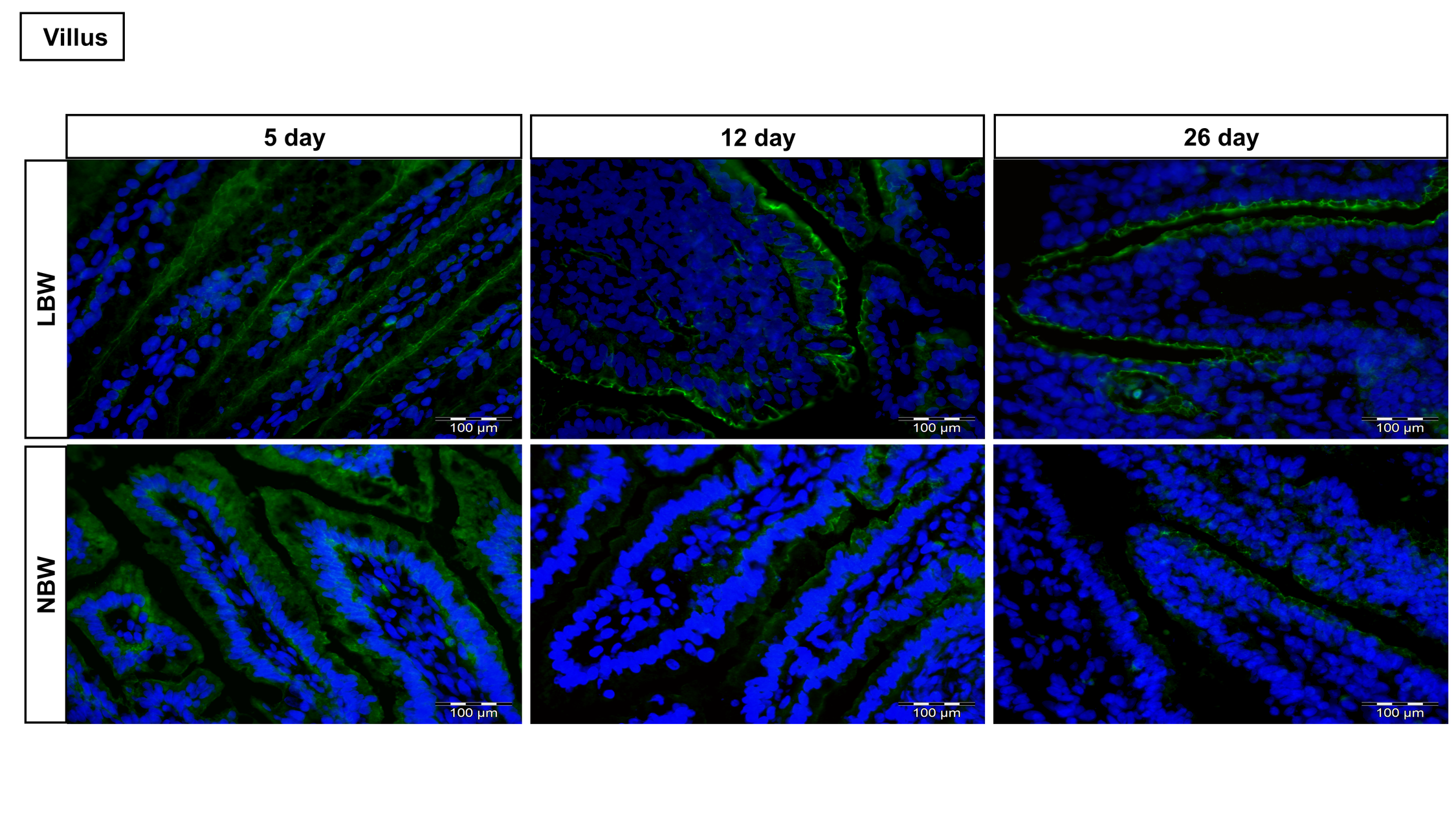

Supplement: S6 Fig — Immunohistochemical images of tight junction protein 2 (green) in low and normal birthweight piglets in three different age groups (5, 12, 26 days); nuclei were counterstained with Hoechst 33258 (blue), scale bars represent 100 μm. (TIF) [file pone.0296427.s006.tif]

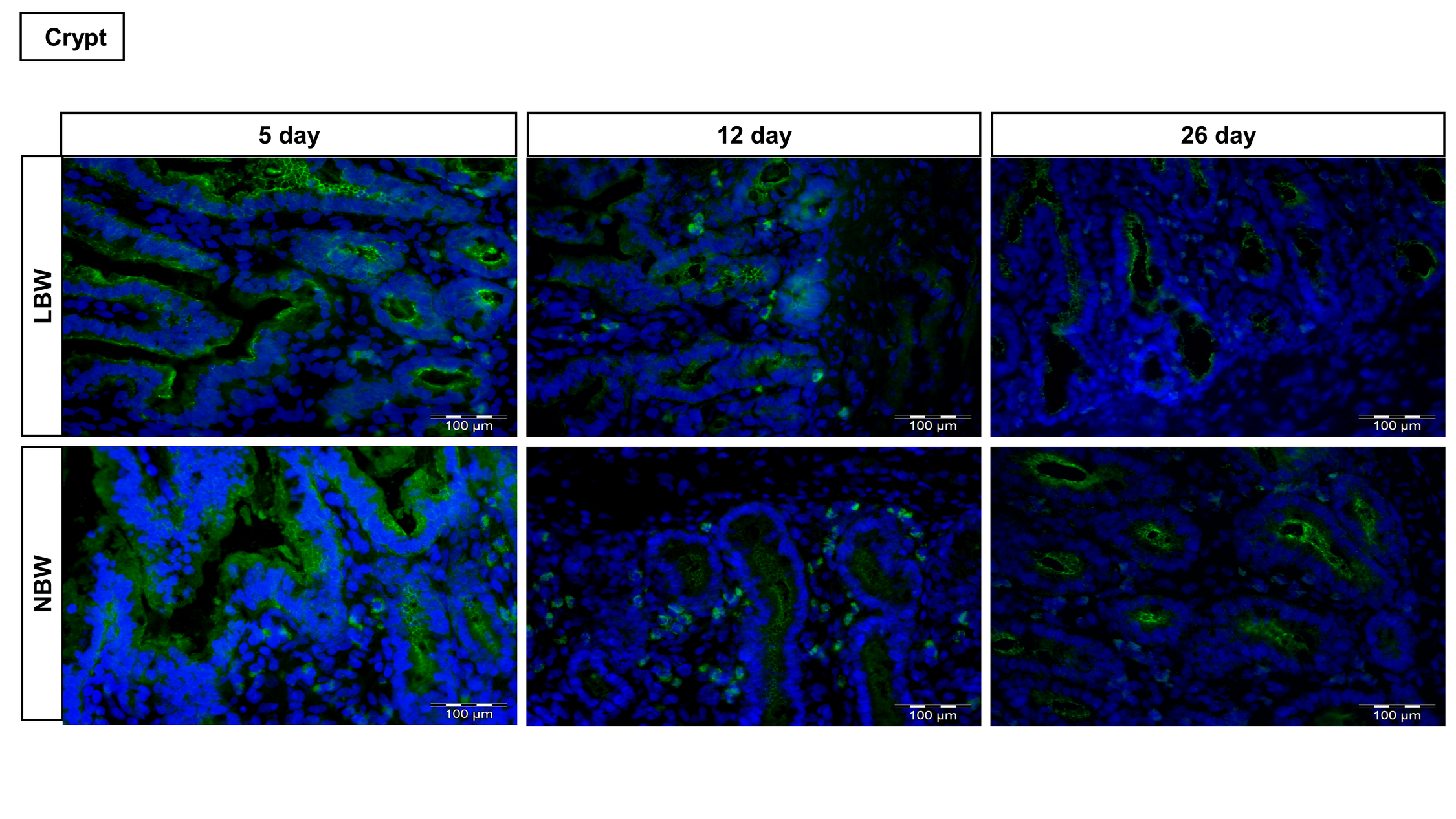

Supplement: S7 Fig — Immunohistochemical images of tight junction protein 2 (green) in low and normal birthweight piglets in three different age groups (5, 12, 26 days); nuclei were counterstained with Hoechst 33258 (blue), scale bars represent 100 μm. (TIF) [file pone.0296427.s007.tif]

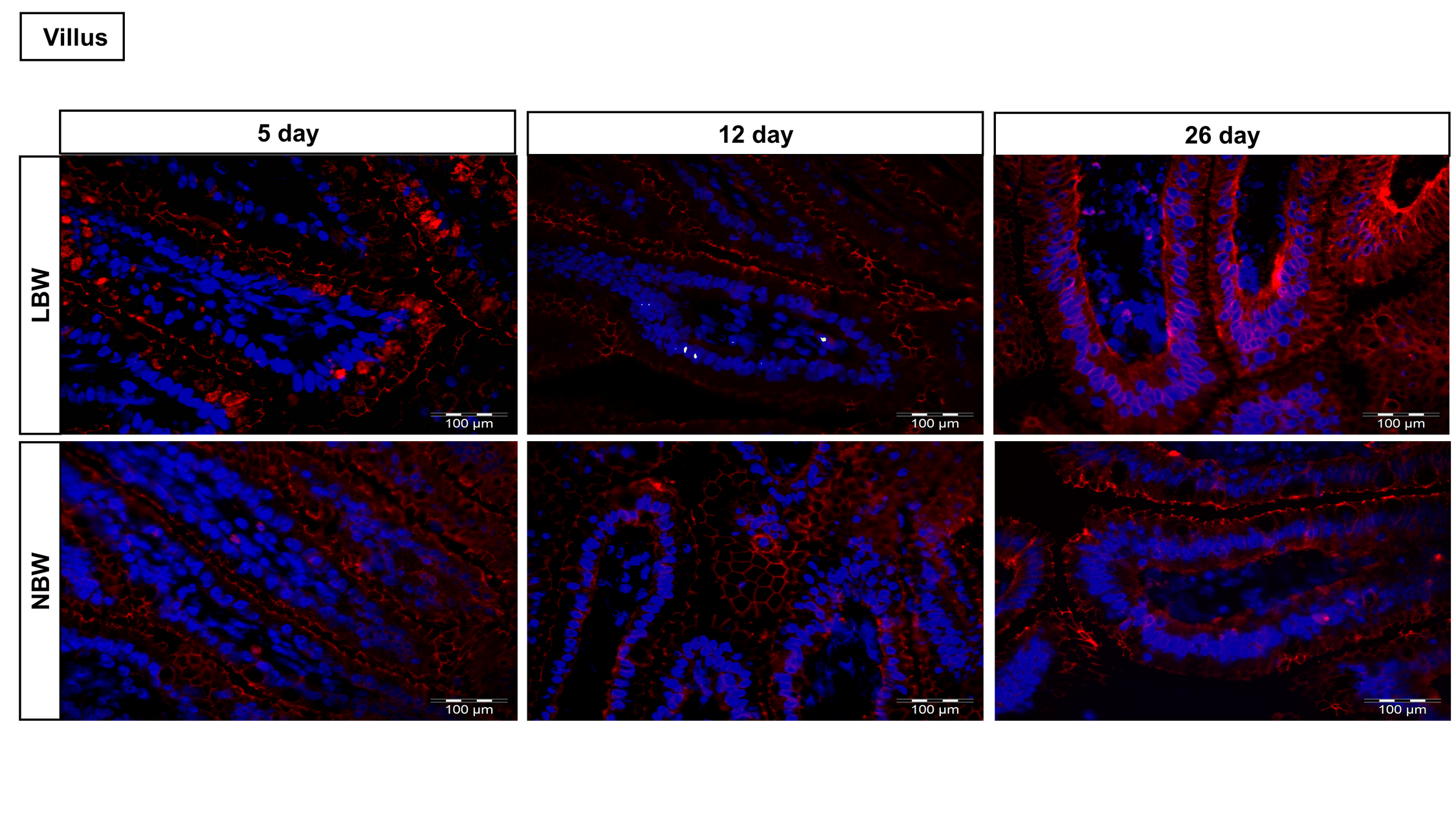

Supplement: S8 Fig — Immunohistochemical images of claudin-4 (red) in low and normal birthweight piglets in three different age groups (5, 12, 26 days); nuclei were counterstained with Hoechst 33258, scale bars represent 100 μm. (TIF) [file pone.0296427.s008.tif]

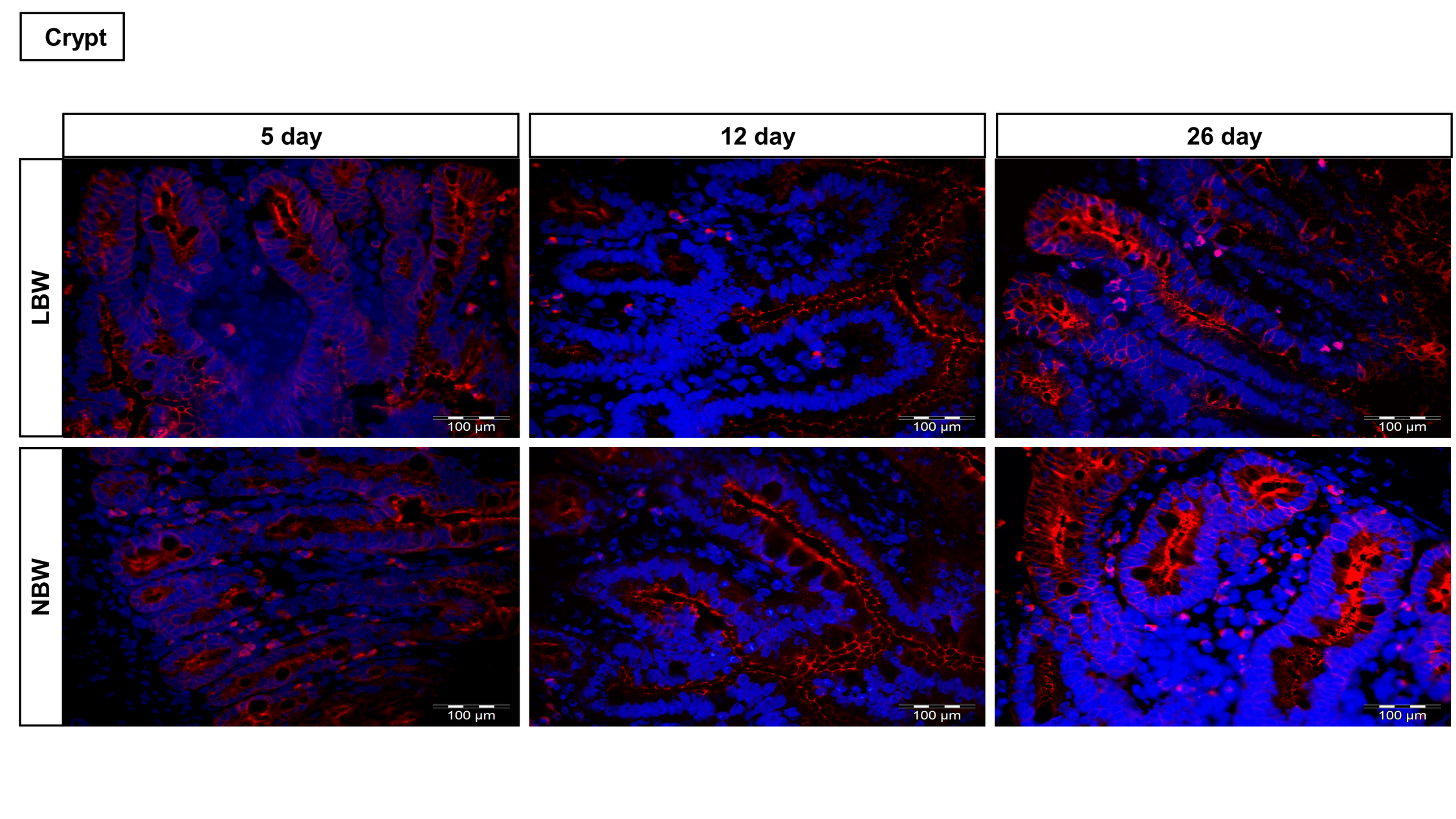

Supplement: S9 Fig — Immunohistochemical images of claudin-4 (red) in low and normal birthweight piglets in three different age groups (5, 12, 26 days); nuclei were counterstained with Hoechst 33258, scale bars represent 100 μm. (TIF) [file pone.0296427.s009.tif]

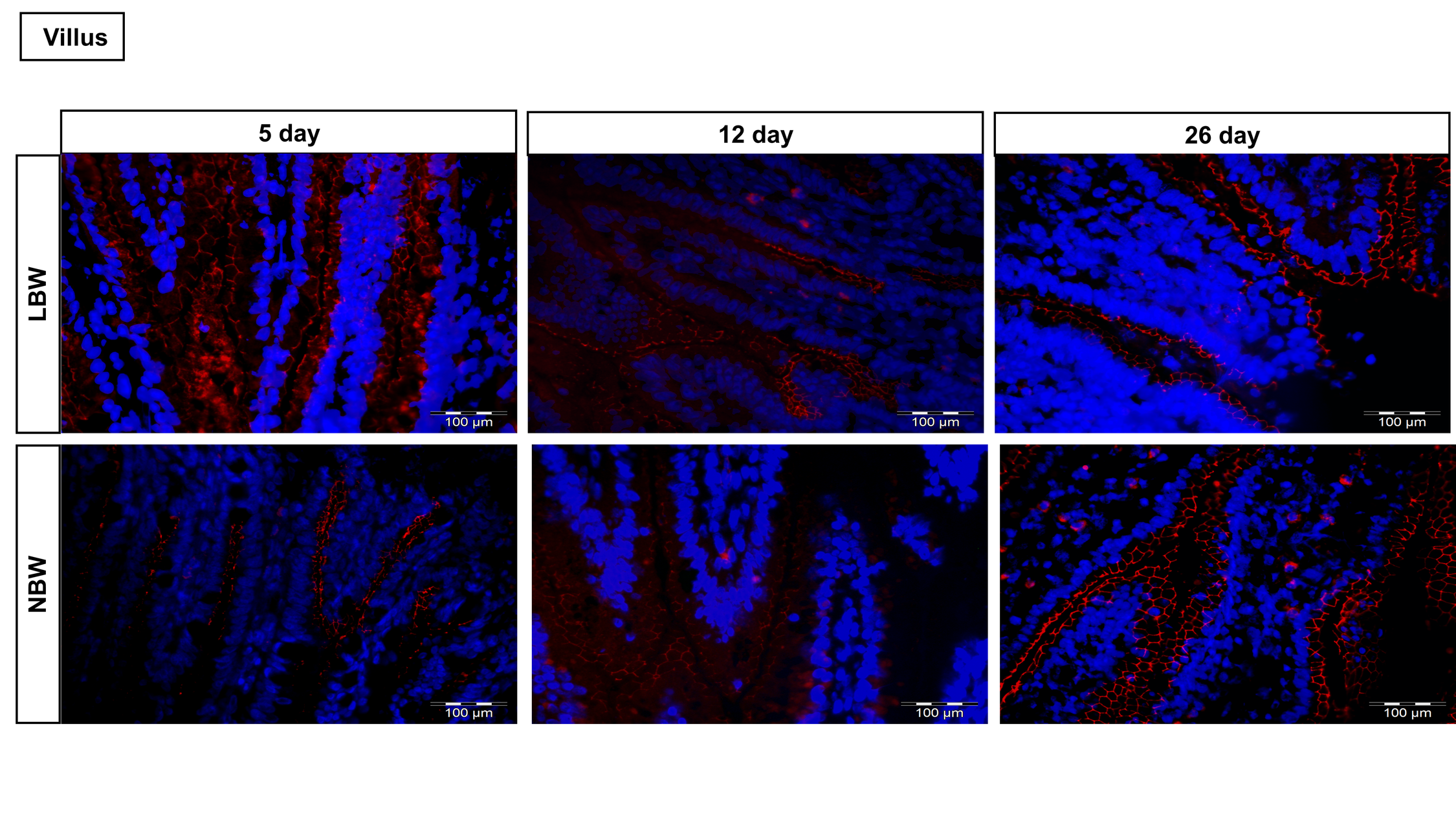

Supplement: S10 Fig — Immunohistochemical images of occludin (red) in low and normal birthweight piglets in three different age groups (5, 12, 26 days); nuclei were counterstained with Hoechst 33258 (blue), scale bars represent 100 μm. (TIF) [file pone.0296427.s010.tif]

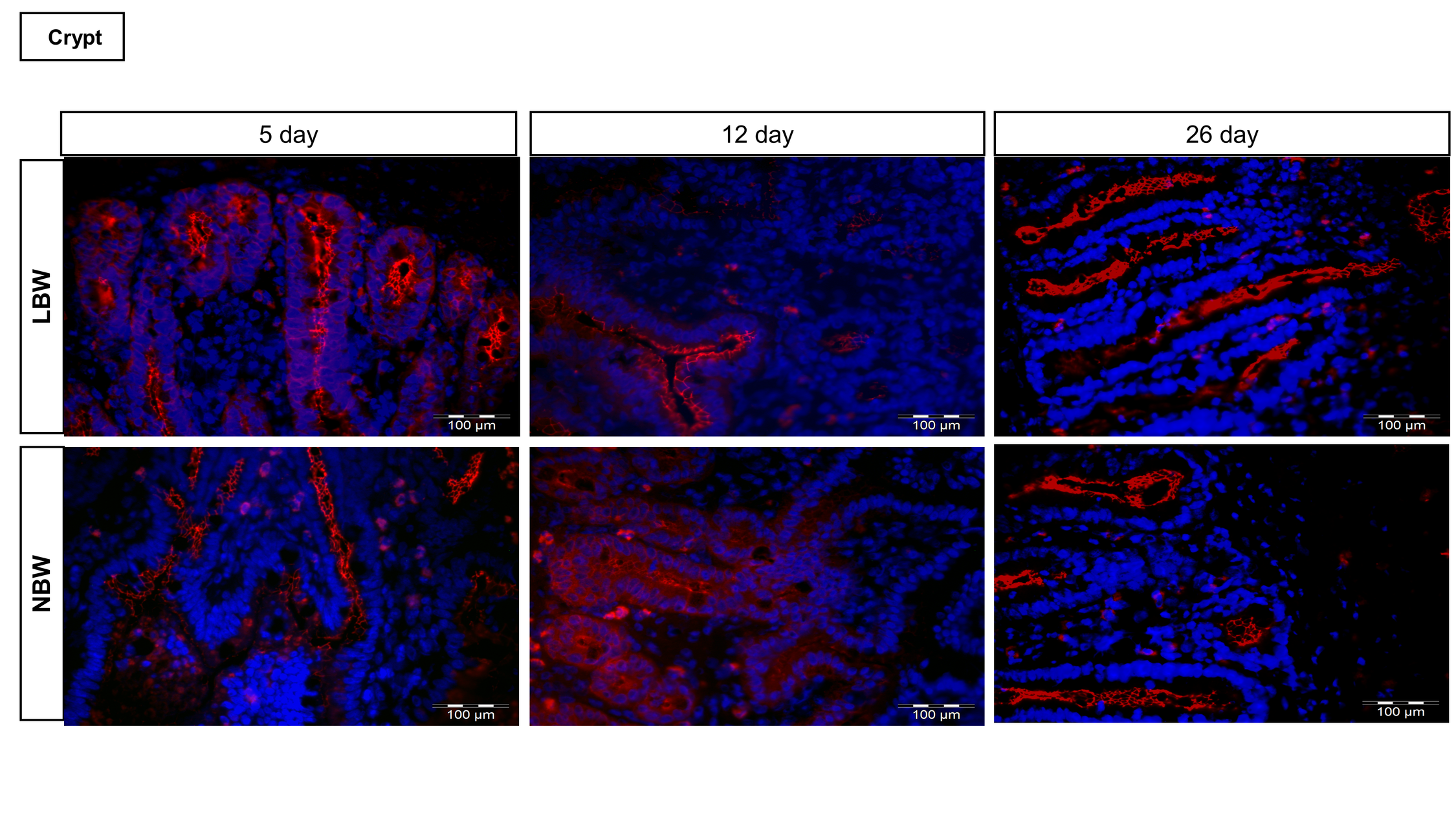

Supplement: S11 Fig — Immunohistochemical images of occludin (red) in low and normal birthweight piglets in three different age groups (5, 12, 26 days); nuclei were counterstained with Hoechst 33258 (blue), scale bars represent 100 μm. (TIF) [file pone.0296427.s011.tif]
