# Supplementary material for: Acute and persistent effects of oral glutamine supplementation on growth, cellular proliferation, and tight junction protein transcript abundance in jejunal tissue of low and normal birthweight pre-weaning piglets
Source: PLoS One. 2024 Jan 2;19(1):e0296427. doi: 10.1371/journal.pone.0296427 (PMC10760696; doi:10.1371/journal.pone.0296427)
Supplement: S1 Table — (DOCX) [file pone.0296427.s012.docx]

PLOS ONE

Acute and persistent effects of oral glutamine supplementation on growth, cellular proliferation, and tight junction protein transcript abundance in jejunal tissue of low and normal birthweight pre-weaning piglets

Johannes Schregel, Johannes Schulze Holthausen, Miriama Sciascia, Solvig Görs, Zeyang Li, Armin Tuchscherer, Elke Albrecht, Jürgen Zentek, Cornelia C. Metges

**S1 Table. Nutrient composition of supplementary creep feed**

| Analyzed ingredients (%) ^1^ |  |
| --- | --- |
| Metabolisable energy, MJ/kg | 13.8 |
| Crude protein | 18.0 |
| Crude fat | 5.3 |
| Crude fiber | 4.0 |
| Crude ash | 5.5 |
| Lysine | 1.35 |
| Methionine | 0.48 |
| Calcium | 0.75 |
| Phosphor | 0.58 |
| Sodium | 0.25 |

Porcistart, Trede & and von Pein GmbH, Itzehoe, Germany

^1^ Standardized to a dry matter content of 88%.
