# Supplementary material for: Acute and persistent effects of oral glutamine supplementation on growth, cellular proliferation, and tight junction protein transcript abundance in jejunal tissue of low and normal birthweight pre-weaning piglets
Source: PLoS One. 2024 Jan 2;19(1):e0296427. doi: 10.1371/journal.pone.0296427 (PMC10760696; doi:10.1371/journal.pone.0296427)
Supplement: S2 Table — (DOCX) [file pone.0296427.s013.docx]

PLOS ONE

Acute and persistent effects of oral glutamine supplementation on growth, cellular proliferation, and tight junction protein transcript abundance in jejunal tissue of low and normal birthweight pre-weaning piglets

Johannes Schregel, Johannes Schulze Holthausen, Miriama Sciascia, Solvig Görs, Zeyang Li, Armin Tuchscherer, Elke Albrecht, Jürgen Zentek, Cornelia C. Metges

**S2 Table.** **Primer sequences**

| Gene | Ref Seq |  | Product | Age |  | Cq |
| --- | --- | --- | --- | --- | --- | --- |
| Name | ID | Direction and Sequence^1^ | Size (bp) | (days) | Efficiency | (mean) |
| Tight junction proteins | | | | | | |
| Tight junction protein 1 | XM_021098827.1 | F’-GACCAACGTAGCTCTGGCAT  R’-TGGACATAGCCTCATTCGCA | 192 | 5 | 1.88 | 25.8 |
|  |  |  |  | 12 | 1.85 | 25.4 |
|  |  |  |  | 26 | 1.88 | 24.4 |
| Tight junction protein 2 | XM_005660143.3 | F’-TAAAGATGGCAACCTGCACGA  R’-TGATGAGCGTCTGCTTGCTG | 155 | 5 | 1.85 | 24.9 |
|  |  |  |  | 12 | 1.84 | 27.0 |
|  |  |  |  | 26 | 1.89 | 25.5 |
| Claudin 4 | NM_001161637.1 | F’- CAACTGCGTGGATGATGAGA  R’-CCAGGGGATTGTAGAAGTCG | 140 | 5 | 1.86 | 24.9 |
|  |  |  |  | 12 | 1.89 | 25.4 |
|  |  |  |  | 26 | 1.84 | 24.8 |
| Occludin | XM_005672522.3 | F’- ACGAGCTGGAGGAAGACTGGATC  R’-GATCCCTTAACTTGCTTCAGTCTATTG | 241 | 5 | 1.88 | 21.2 |
|  |  |  |  | 12 | 1.86 | 26.8 |
|  |  |  |  | 26 | 1.86 | 24.0 |
| Cellular proliferation and apoptosis | | | | | | |
| Proliferating Cell Nuclear Antigen | NM_001291925.1 | F’-GTTGTCACAAACGAGTAATGTTGA  R’-GCTGAACTGGTTCATTCATCTCT | 71 | 5 | 1.88 | 25.8 |
|  |  |  |  | 12 | 1.88 | 26.4 |
|  |  |  |  | 26 | 1.95 | 21.1 |
| Caspase-3 | NM_214131.1 | F’-GAACTCTAACTGGCAAACCCA  R’-ACGCCATGTCATCTTCAGTC | 105 | 5 | 1.92 | 23.4 |
|  |  |  |  | 12 | 1.91 | 27.1 |
|  |  |  |  | 26 | 1.93 | 25.7 |
| Reference genes | | | | | | |
| Actin-beta | XM_021086047.1 | F’-CACGCCATCCTGCGTCTGGA  R’-AGCACCGTGTTGGCGTAGAG | 100 | 5 | 1.91 | 18.6 |
|  |  |  |  | 12 | 1.74 | 25.8 |
|  |  |  |  | 26 | 1.83 | 22.8 |
| Hypoxanthine Phosphoribosyltransferase 1 | XM_021079503.1 | F’-GGACTTGAATCATGTTTGTG  R’-CAGATGTTTCCAAACTCAAC | 91 | 5 | 1.87 | 27.2 |
|  |  |  |  | 12 | 1.82 | 28,.0 |
|  |  |  |  | 26 | 1.87 | 21.8 |
| Peptidylprolyl isomerase A | NM_214353 | F’-AGCACTGGGGAGAAAGGATT  R’-TCCAAGTCTTCACGGAGTTTGTT | 220 | 5 | 1.90 | 18.7 |
|  |  |  |  | 12 | 1.89 | 21.3 |
|  |  |  |  | 26 | 1.82 | 21.3 |
| Ribosomal protein S18 | NM_213940.1 | F’-GGATGTGAAGGATGGGAAGTACA  R’-TCCAAGTCTTCACGGAGTTTGTT | 72 | 5 | 1.89 | 22.8 |
|  |  |  |  | 12 | 1.89 | 21.0 |
|  |  |  |  | 26 | 1.88 | 17.0 |
| Topoisomerase 2-beta | NM_001258386.1 | F’- CCCAGTTGGCTGGATCTGTT  R’-ATAACGAGGGCTTGCAGCAT | 182 | 5 | 1.91 | 25.9 |
|  |  |  |  | 12 | 1.90 | 25.5 |
|  |  |  |  | 26 | 1.86 | 20.9 |

^1^ Caspase-3, Claudin 4, Hypoxanthine Phosphoribosyltransferase 1, Occludin, Proliferating Cell Nuclear Antigen, Tight junction protein 1, Tight junction protein 2, were designed for this study, Actin-beta from Nygard et al., 2007 [1], Peptidylprolyl isomerase A and Ribosomal protein S18 from Rasch et al., 2019 [2], and Topoisomerase 2-beta from Schregel et al., 2022 [3]

Supplementary References

1. Nygard AB, Jorgensen CB, Cirera S, Fredholm M. Selection of reference genes for gene expression studies in pig tissues using SYBR green qPCR. BMC Mol. Biol.. 2007;8:67. Epub 2007/08/19. doi: 10.1186/1471-2199-8-67. PubMed PMID: 17697375; PubMed Central PMCID: PMCPMC2000887.

2. Rasch I, Gors S, Tuchscherer A, Viergutz T, Metges CC, Kuhla B. Substitution of Dietary Sulfur Amino Acids by dl-2-Hydroxy-4-Methylthiobutyric Acid Reduces Fractional Glutathione Synthesis in Weaned Piglets. J. Nutr.. 2019. Epub 2019/11/28. doi: 10.1093/jn/nxz272. PubMed PMID: 31773161.

3. Schregel J, Schulze Holthausen J, Sciascia M, Li Z, Gors S, Eggert A, et al. Effects of oral glutamine supplementation on jejunal morphology, development, and amino acid profiles in male low birth weight suckling piglets. Plos One. 2022;17(4):e0267357. Epub 2022/04/28. doi: 10.1371/journal.pone.0267357. PubMed PMID: 35476806; PubMed Central PMCID: PMCPMC9045636.
